# Site- and horizon-specific patterns of microbial community structure and enzyme activities in permafrost-affected soils of Greenland

**DOI:** 10.3389/fmicb.2014.00541

**Published:** 2014-10-16

**Authors:** Antje Gittel, Jiří Bárta, Iva Kohoutová, Jörg Schnecker, Birgit Wild, Petr Čapek, Christina Kaiser, Vigdis L. Torsvik, Andreas Richter, Christa Schleper, Tim Urich

**Affiliations:** ^1^Department of Biology, Centre for Geobiology, University of BergenBergen, Norway; ^2^Department of Bioscience, Center for Geomicrobiology, Aarhus UniversityAarhus, Denmark; ^3^Department of Ecosystems Biology, University of South BohemiaČeské Budějovice, Czech Republic; ^4^Division of Terrestrial Ecosystem Research, Department of Microbiology and Ecosystem Science, University of ViennaVienna, Austria; ^5^Austrian Polar Research InstituteVienna, Austria; ^6^Division of Archaea Biology and Ecogenomics, Department of Ecogenomics and Systems Biology, University of ViennaVienna, Austria

**Keywords:** climate change, extracellular enzyme activities, Greenland, permafrost-affected soils, microbial communities

## Abstract

Permafrost-affected soils in the Northern latitudes store huge amounts of organic carbon (OC) that is prone to microbial degradation and subsequent release of greenhouse gasses to the atmosphere. In Greenland, the consequences of permafrost thaw have only recently been addressed, and predictions on its impact on the carbon budget are thus still highly uncertain. However, the fate of OC is not only determined by abiotic factors, but closely tied to microbial activity. We investigated eight soil profiles in northeast Greenland comprising two sites with typical tundra vegetation and one wet fen site. We assessed microbial community structure and diversity (SSU rRNA gene tag sequencing, quantification of bacteria, archaea and fungi), and measured hydrolytic and oxidative enzyme activities. Sampling site and thus abiotic factors had a significant impact on microbial community structure, diversity and activity, the wet fen site exhibiting higher potential enzyme activities and presumably being a hot spot for anaerobic degradation processes such as fermentation and methanogenesis. Lowest fungal to bacterial ratios were found in topsoils that had been relocated by cryoturbation (“buried topsoils”), resulting from a decrease in fungal abundance compared to recent (“unburied”) topsoils. *Actinobacteria* (in particular *Intrasporangiaceae*) accounted for a major fraction of the microbial community in buried topsoils, but were only of minor abundance in all other soil horizons. It was indicated that the distribution pattern of *Actinobacteria* and a variety of other bacterial classes was related to the activity of phenol oxidases and peroxidases supporting the hypothesis that bacteria might resume the role of fungi in oxidative enzyme production and degradation of phenolic and other complex substrates in these soils. Our study sheds light on the highly diverse, but poorly-studied communities in permafrost-affected soils in Greenland and their role in OC degradation.

## Introduction

It has been predicted that due to the strong increase in mean annual surface air temperature (up to 8°C), 25% of the arctic permafrost could thaw until the end of this century (IPCC, 2007). Increased active layer depths and longer frost-free vegetation periods are predicted to accelerate the microbial decomposition of soil organic matter (SOM) and, together with higher nutrient availability, may generate a positive feedback to climate warming (Biasi et al., 2008; Schuur et al., 2008; Tarnocai et al., 2009; Schuur and Abbott, 2011; MacDougall et al., 2012). In addition to abiotic factors such as elevated temperatures, changes in hydrology, moisture, quantity and quality of the SOM, the extent of this feedback will greatly depend on the response of the microbial communities as the main biotic drivers of biogeochemical processes (Singh et al., 2010; Xu et al., 2011; Graham et al., 2012).

The diversity of microbial communities in permafrost-affected soils, their functional potential and the coupling of SOM properties and microbial activity have been increasingly recognized and addressed in several studies in Siberia (Ganzert et al., 2007; Liebner et al., 2008; Gittel et al., 2014; Schnecker et al., 2014), the Canadian Arctic (Yergeau et al., 2010; Frank-Fahle et al., 2014), Alaska (Mackelprang et al., 2011; Lipson et al., 2013; Tas et al., 2014), and Svalbard (Tveit et al., 2012, 2014; Alves et al., 2013). High-arctic permafrost regions in Greenland, however, have received only little attention so far, despite the recognized relevance of thawing permafrost and the potential decomposition of organic carbon (OC) stocks (Masson-Delmotte et al., 2012; Elberling et al., 2013; Ganzert et al., 2014). The top permafrost is thawing at present (more than 1 cm per year) and model simulations predicted an increase of active layer depth by 35 cm as a result of 6°C warming in the next 70 years (Hollesen et al., 2011; Masson-Delmotte et al., 2012). Data on the amount of OC stored in soils in the high Arctic permafrost regions of Greenland are still incomprehensive and, as recent studies suggested, the total OC pool in these regions is probably underestimated (Horwath Burnham and Sletten, 2010; Hugelius et al., 2013). Cryoturbation, the burial of topsoil material into deeper soil horizons by repeated freeze–thaw events, is an important storage mechanism for SOM (Kaiser et al., 2007). Very little information is available on OC stocks in cryoturbated soil pockets in Greenland (Palmtag, 2011), but globally they contain more than one third of the total OC stored in arctic soils (Tarnocai et al., 2009; Harden et al., 2012). We recently showed that microbial communities in cryoturbated material were not adapted to the available substrate having a potentially restraining effect on enzyme activities (Gittel et al., 2014; Schnecker et al., 2014). This decoupling of microbial community composition from SOM properties might explain the persistence of OC in buried topsoils, but also implies that a shift in community composition and enhanced microbial activity in cryoturbated soil pockets would reinforce a positive feedback to climate change.

In this study, we sampled organic and mineral topsoils, mineral subsoils, and buried topsoils from the active layer as well as frozen mineral soils (hereafter called permafrost samples) at three sites in northeastern Greenland. We aimed to (1) explore the microbial diversity and community composition, (2) determine potential activities of oxidative and hydrolytic extracellular enzymes involved in the degradation of organic matter, and (3) assess possible correlations between enzyme activities and shifts in community composition. We hypothesized that different sampling sites and/or soil horizons harbor distinct communities and that changes in community structure are reflected in the enzymatic and thus the degradation potential of these communities. We were particularly interested in the distribution patterns of both fungal and bacterial decomposers (e.g., actinobacteria) that have previously been identified as critical components in the delayed decomposition of organic matter in cryoturbated soils (Gittel et al., 2014).

## Materials and methods

### Field site description and soil sampling

Soils were sampled in close vicinity to the Zackenberg Research Station, northeast Greenland (74°28′N, 20°32′W). The climate is characterized by a mean annual air temperature of around −10°C and an annual precipitation of about 150 mm. Minimum air temperatures are below −40°C, and sub-surface temperatures (5 cm below the surface) are below −18° for about 4 months per year (Elberling and Brandt, 2003). Samples were taken from 3 different sampling sites (Table S1, Figure S1). Sites 1 and 3 were characterized by frost boils and earth hummocks and dominated by a diverse community of typical tundra vegetation (e.g., *Salix arctica, Vaccinium uliginosum*). Site 2 was a wet fen dominated by mosses and grasses (e.g., *Eriophorum angustifolium*). Two to three replicate plots that were distributed within an area of approximately 50 × 50 m were sampled at each sampling site (site 1: plots A–C, site 2: plots D–F, and site 3: plots G,H; Figure S1). For each plot, 3–5 replicate soil cores were collected close to each other (max. area: 1 × 1 m) to obtain enough material from each soil horizon. Main soil horizons were identified and pooled to obtain one replicate sample. Soil material was sampled from the active layer and the permafrost layer (PF), resulting in a total of 37 soil samples (Table S2). Soil classification follows the USDA Soil Taxonomy (Soil Survey Staff, 2010). Samples from the active layer included organic and mineral topsoil horizons (O and A), mineral subsoil horizons (B), and topsoil material that was buried into deeper soil horizons by cryoturbation (Ojj and Ajj, collectively called “J” in the following). Initial soil processing included removal of living roots prior to homogenizing the soil fraction of each sample. Samples for extracting total nucleic acids were fixed in RNA*later* RNA Stabilization Reagent (Ambion Inc., Life Technologies) and kept cold until further processing. Samples for the analyses of soil properties, microbial biomass and potential enzyme activities were stored in closed polyethylene bags at 4°C until analyzed.

### Soil properties, microbial biomass and extracellular enzyme activities

Soil water content was estimated by drying the soil over night at 60°C and reweighing the samples. Total organic carbon (TOC) and total nitrogen (TN) contents were determined in dried (60°C) and ground samples with an EA IRMS system (EA 1110, CE Instruments, Milan, Italy, coupled to a Finnigan MAT Delta Plus IRMS, Thermo Fisher Scientific). Microbial C and N were determined using chloroform-fumigation extraction (Kaiser et al., 2010). Fumigated and non-fumigated soil samples were extracted with 0.5 M K_2_SO_4_ and analyzed for extracted C and N on a TOC/TN analyzer (LiquicTOC II, Elementar, Germany). Microbial C and N were calculated as the difference in C and N concentrations between fumigated and non-fumigated samples.

Potential extracellular hydrolytic and oxidative enzyme activities involved in the degradation of organic macromolecules (namely cellulose, chitin, peptides, lignin) were measured according to Kaiser et al. (2010) using microplate fluorometric and photometric assays. The enzymes under study, their acronyms, function and substrates are listed in Table 1. One gram of sieved soil was suspended in 100 ml sodium acetate buffer (100 mM, pH 5.5) and ultra-sonicated at low-energy. Potential activities of 1,4-β-cellobiohydrolase (CBH), 1,4-β-poly-N-acetylglucosaminidase (chitotriosidase, CHT), β-N-acetylglucosaminidase (NAG), and leucine aminopeptidase (LAP) were measured fluorometrically using 4-methylumbelliferyl- (MUF) and aminomethylcoumarin- (AMC) substrates (Marx et al., 2001; Kaiser et al., 2010). 200 μL of the soil suspension and 50 μL substrate (MUF-β-D-cellobioside, MUF-N-acetyl-β-D-glucosaminide, MUF-β-D-N,N′,N″-triacetylchitotrioside, and L-leucine-7-amido-4-methyl coumarin, respectively) were pipetted into black microtiter plates in 5 analytical replicates. For each sample, a standard curve with methylumbelliferyl was used for calibration of cellobiosidase, N-acetylglucosaminidase and chitotriosidase, whereas aminomethylcoumarin was used for calibration of leucine amino-peptidase. Plates were incubated for 140 min in the dark and fluorescence was measured at 450 nm emission at an excitation of 365 nm (Tecan Infinite M200 fluorimeter). Potential phenol oxidase (POX) and peroxidase (PER) activities were measured photometrically using the L-3,4-dihydroxyphenylalanin (L-DOPA) assay (Sinsabaugh et al., 1999; Kaiser et al., 2010). Subsamples were taken from the soil suspension (see above) and mixed with a 20 mM L-DOPA solution (1:1). Samples were shaken for 10 min, centrifuged and aliquotes were pipetted into microtiter plates (6 analytical replicates per sample). Half of the wells additionally received 10 μL of a 0.3% H_2_O_2_ solution for measurement of peroxidase. Absorption was measured at 450 nm at the starting time point and after 20 h. Enzyme activity was calculated from the difference in absorption between the two time points.

**Table 1:** **Hydrolytic and oxidative extracellular enzymes assayed**.

**Enzyme**	**Acronym**	**Type/Function**	**Substrate**	**EC number**
1,4-β-cellobiohydrolase	CBH	Hydrolytic, C-acquiring	Cellulose	3.2.1.91
1,4-β-poly-N-acetylglucosaminidase (chitotriosidase)^a^	CHT	Hydrolytic, N-acquiring (also liberates C)	Chitin, peptidoglycan	3.2.1.14
β-N-acetylglucosaminidase (exochitinase)^a^	NAG	Hydrolytic, N-acquiring (also liberates C)	Chitin, peptidoglycan	3.2.1.52
Leucine aminopeptidase	LAP	Hydrolytic, N-acquiring	Peptides	3.4.11.1
Phenoloxidase	POX	Oxidative, C- and N-acquiring	Lignin, phenolic and other complex compounds	1.10.3.2
Peroxidase	PER	Oxidative, C- and N-acquiring	Lignin, phenolic and other complex compounds	1.11.1.7

a *Chitotriosidase activity is defined as the random cleavage at internal points in the chitin or peptidoglycan chain. Exochitinase activity is defined as the progressive action starting at the non-reducing end of chitin or peptidoglycan with the release of chitobiose or N-acetyl-glucosamine units*.

### Nucleic acid preparation for quantitative PCR

Nucleic acid extractions were conducted according to a modified bead-beating protocol (Urich et al., 2008). Soil samples were washed with DEPC-PBS (Diethylpyrocarbonate, 0.1%; 1 × phosphate-buffered saline) to remove the RNA*later*. Approximately 0.5 g of soil was added to a FastPrep™ Lysis Matrix E tube (MP Biomedicals, Solon, OH, USA). Hexadecyltrimethylammonium bromide (CTAB) extraction buffer containing 5% CTAB (in 0.7 M NaCl, 120 mM potassium phosphate, pH 8.0) and 0.5 mL phenol-chloroform-isoamylalcohol (25:24:1) was added and shaken in a FastPrep Instrument (MP Biomedicals, Solon, OH, USA) at speed 5–6 for 45 s. After bead beating, the samples were extracted with chloroform and precipitated in a PEG 6000/1.6 M NaCl solution. Pellets were washed with 70% ethanol and re-suspended in molecular biology grade water. Nucleic acids were further purified using the CleanAll DNA/RNA Clean-up and Concentration Micro Kit (Norgen Biotek Corp., Ontario, Canada). Total DNA was quantified using SybrGreen (Leininger et al., 2006).

### Quantification of SSU rRNA genes by quantitative PCR

Bacterial, archaeal and fungal SSU rRNA genes were amplified as described previously (Gittel et al., 2014). Briefly, bacterial and archaeal SSU rRNA genes were amplified with the primer set Eub338Fabc/Eub518R (Daims et al., 1999; Fierer et al., 2005) for *Bacteria* and Arch519F/Arch908R (Jurgens et al., 1997; Teske and Sorensen, 2007) for *Archaea*. Fungal SSU genes were amplified using the primers nu-SSU-0817-5′ and nu-SSU1196-3′ (Borneman and Hartin, 2000). Product specificity was confirmed by melting point analysis and amplicon size was verified with agarose gel electrophoresis. Bacterial and archaeal DNA standards consisted of a dilution series (ranging from 10^0^ to 10^8^ gene copies μL^−1^) of a known amount of purified PCR product obtained from genomic *Escherichia coli* DNA and *Natrialba magadii* DNA by using the SSU gene-specific primers 8F/1492R and 21F/1492R, respectively (DeLong, 1992; Loy et al., 2002). Fungal DNA standards consisted of a dilution series (ranging from 10^0^ to 10^7^ gene copies μL^−1^) of a known amount of purified PCR product obtained from genomic *Fusarium oxysporum* DNA by using the SSU gene-specific primers nu-SSU-0817-5′ and nu-SSU1196-3′ (Borneman and Hartin, 2000). Samples, standards and non-template controls were run in triplicates. Detection limits for the assays (i.e., lowest standard concentration that is significantly different from the non-template controls) were less than 100 gene copies for each of the genes. For statistical analyses (calculation of means and deviations per site, One-Way ANOVA), samples below the detection limit have been assigned a value of half the detection limit of the respective assay.

### Nucleic acid preparation and barcoded amplicon sequencing

Nucleic acid extration and bacterial and archaeal SSU rRNA amplification were performed according to standardized protocols of the Earth Microbiome Project (www.earthmicrobiome.org/emp-standard-protocols). Amplicons were sequenced on the Illumina MiSeq platform (Caporaso et al., 2011, 2012). Sequence data along with MiMARKs compliant metadata are available from the Qiime database (http://www.microbio.me/emp/, study no. 1034).

### Sequence analysis

Quality filtering of reads was applied as described previously (Caporaso et al., 2011). Reads were truncated at their first low-quality base (defined by an “A” or “B” quality score). Reads shorter than 75 bases were discarded, as were reads whose barcode did not match an expected barcode. Reads were assigned to operational taxonomic units (OTUs) using closed-reference OTU picking protocol in QIIME version 1.7.0 (Caporaso et al., 2010), with uclust (Edgar, 2010) being applied to search sequences against a subset of the Greengenes database version 13_5 (DeSantis et al., 2006) with reference sequences clustered at 97% sequence identity. Reads were assigned to OTUs based on their best hit to this database at greater than or equal to 97% sequence identity. Reads without a hit were discarded. A major advantage of this procedure is that the OTUs are defined by trusted reference sequences. It furthermore serves as a quality control filter: erroneous reads will likely be discarded as not hitting the reference data set. The primary disadvantage is that sequences that are not already known (i.e., represented in the reference data set) will be excluded. Taxonomy was assigned by accepting the Greengenes taxonomy string of the best matching Greengenes sequence. Alpha and beta diversity analyses were performed on data rarefied to 17,000 sequences per sample. Principal coordinates analysis (PCoA) was used to visualize differences between sampling sites and soil horizons using unweigthed Unifrac distances, a distance measure between communities using phylogenetic information (Lozupone and Knight, 2005). To assess differences in community structure between sites, soil horizons and replicate pits, a matrix of Bray-Curtis distances of square-root transformed relative abundances of each OTU was used for permutational ANOVA (PERMANOVA). Alpha diversity measures (richness, Shannon and inverse Simpson indices and evenness) were calculated in R 2.15.0 (R Development Core Team, 2012) using the packages vegan (Oksanen et al., 2007) and fossil (Vavrek, 2011). To test for the presence of a core prokaryotic community and distinct phylotypes in buried topsoils, we acquired the core OTUs being present in all buried topsoil samples on the non-rarified data set using the compute_core_microbiome function in Qiime.

### Further statistical analyses

All analyses were performed in R 2.15.0 (R Development Core Team, 2012) using the packages vegan (Oksanen et al., 2007), FactoMineR (Husson et al., 2013), and missMDA (Josse and Husson, 2013). Prior to One-Way ANOVA, Shapiro-Wilk tests and Bartlett tests were used to examine whether the conditions of normality and homogeneity of variance were met by non-transformed data sets as well as transformed data sets (log transformation: soil properties, biomass and gene abundance variables, square-root transformation: OTU abundance data). One-Way ANOVA was followed by Tukey's HSD test to determine significant differences between soil horizon (O/A, B, J, and PF) for each site. Differences were considered significant at *P* < 0.05.

Principle component analysis (PCA) was used to summarize data on soil properties and microbial biomass. Data were standardized (zero mean and unit standard deviation), and the number of dimensions were estimated by cross-validation and missing values were imputed using the package missMDA (Josse and Husson, 2013). Canonical correspondence analysis (CCA) was applied to relate microbial activity (potential enzyme activities) to differences in community structure (i.e., changes in relative OTU abundances). Potential enzyme activities were standardized to zero mean and unit standard deviation. Pearson's product–moment correlation (R) was used to assess linkages between individual OTUs, soil properties and enzyme activities.

## Results

### Soil properties and microbial biomass

Soil samples were collected from three different sites in northeast Greenland including two sites with typical tundra vegetation (site 1, *n* = 15 and site 3, *n* = 9), and a wet fen site (site 2, *n* = 13), and from four different soil horizons at each of these sites: organic and mineral topsoil (O and A, *n* = 10), mineral subsoil (B, *n* = 8), buried topsoil (J, *n* = 12), and the permafrost layer (PF, *n* = 7). Topsoil horizons exhibited a higher moisture, TOC and TN content, as well as higher microbial biomass (Cmic, Nmic) compared to buried topsoils, mineral subsoils and permafrost samples (Table 2). Buried topsoils were characterized by higher moisture, TOC, TN, Cmic, and Nmic than the mineral subsoils they were embedded in, although these differences were not significant in all of the sampling sites (Table 2). Moisture, TOC, and TN in topsoil and buried topsoil samples from site 2 were higher than in the corresponding samples from sites 1 and 3. PCA analysis separated O and A horizons along the first principal component and placed J horizons at an intermediate position (Figure S2). B horizons and PF samples were positioned closer to each other, thus representing higher between-sample similarity than O, A and J horizons.

**Table 2:** **Soil properties and microbial biomass^a^**.

**Site**	Soil group^*^	**Number of samples**	**Moisture (%)**	**TOC (% dw)**	**TN (% dw)**	**CN ratio**	**Cmic (μmol g^−1^ dw)**	**Nmic (μmol g^−1^dw)**
1	A	5	45.5±8.8 (a)	12.4±4.2 (a)	0.8±0.2 (a)	14.6±1.1 (a)	161.5±76.2 (a)	15.1±8.7 (a)
	B	3	21.2±4.5 (b)	2.0±0.8 (bc)	0.1±0.1 (bc)	13.6±0.2 (a)	38.3±13.2 (b)	1.8±0.9 (a)
	J	4	36.8±7.1 (ab)	7.6±2.5 (ab)	0.5±0.1 (ab)	14.6±1.9 (a)	59.1±32.1 (ab)	3.1±2.6 (a)
	PF	3	24.8±6.9 (b)	0.7±0.2 (c)	0.04±0.03 (c)	18.8±9.2 (a)	33.1±7.4 (ab)	1.0
2	O	3	77.2±4.2 (a)	29.0±2.4 (a)	1.4±0.1 (a)	20.3±1.4 (a)	175.5 (a)	24.6±20.4 (a)
	B	3	29.2±5.3 (b)	4.5±2.5 (b)	0.3±0.2 (b)	15.6±0.5 (a)	22.6±9.2 (b)	2.0±1.3 (a)
	J	5	70.1±13.0 (a)	17.8±5.4 (c)	1.1±0.5 (a)	18.2±4.3 (a)	62.0±26.2 (b)	3.0±1.3 (a)
	PF	2	33.1±11.9 (b)	2.4±2.3 (b)	0.2±0.1 (b)	13.7±2.6 (a)	10.6±2.8 (b)	3.9
3	O	2	42.9±1.8 (a)	17.5±0.2 (a)	0.8±0.0 (a)	21.3±0.3 (a)	316.0±10.4 (a)	29.6±6.3 (a)
	B	2	20.1±3.1 (b)	2.3±0.8 (b)	0.2±0.1 (b)	14.8±0.3 (b)	33.5±22.2 (b)	1.9±0.7 (b)
	J	3	35.0±5.8 (a)	8.5±2.1 (c)	0.5±0.1 (c)	16.8±1.3 (b)	69.5±63.7 (b)	6.5±7.2 (b)
	PF	2	91.2±0.5 (c)	2.4±0.5 (b)	0.2±0.0 (b)	14.6±1.0 (b)	143.2±118.5 (ab)	10.8 (ab)

a *Means and standard deviations were calculated from soil samples classified into each soil group. Small letters in brackets indicate significant differences as determined by One-Way ANOVA and Tukey's HSD test*.

* *Soil samples were grouped according to their total organic carbon (TOC). For details see Table S2 (Supplementary Material)*.

*TOC, total organic carbon; TN, total nitrogen; CN ratio, carbon-to-nitrogen ratio; Cmic, microbial carbon; Nmic, microbial nitrogen*.

### Abundances of bacteria, archaea and fungi

**Figure 1:**
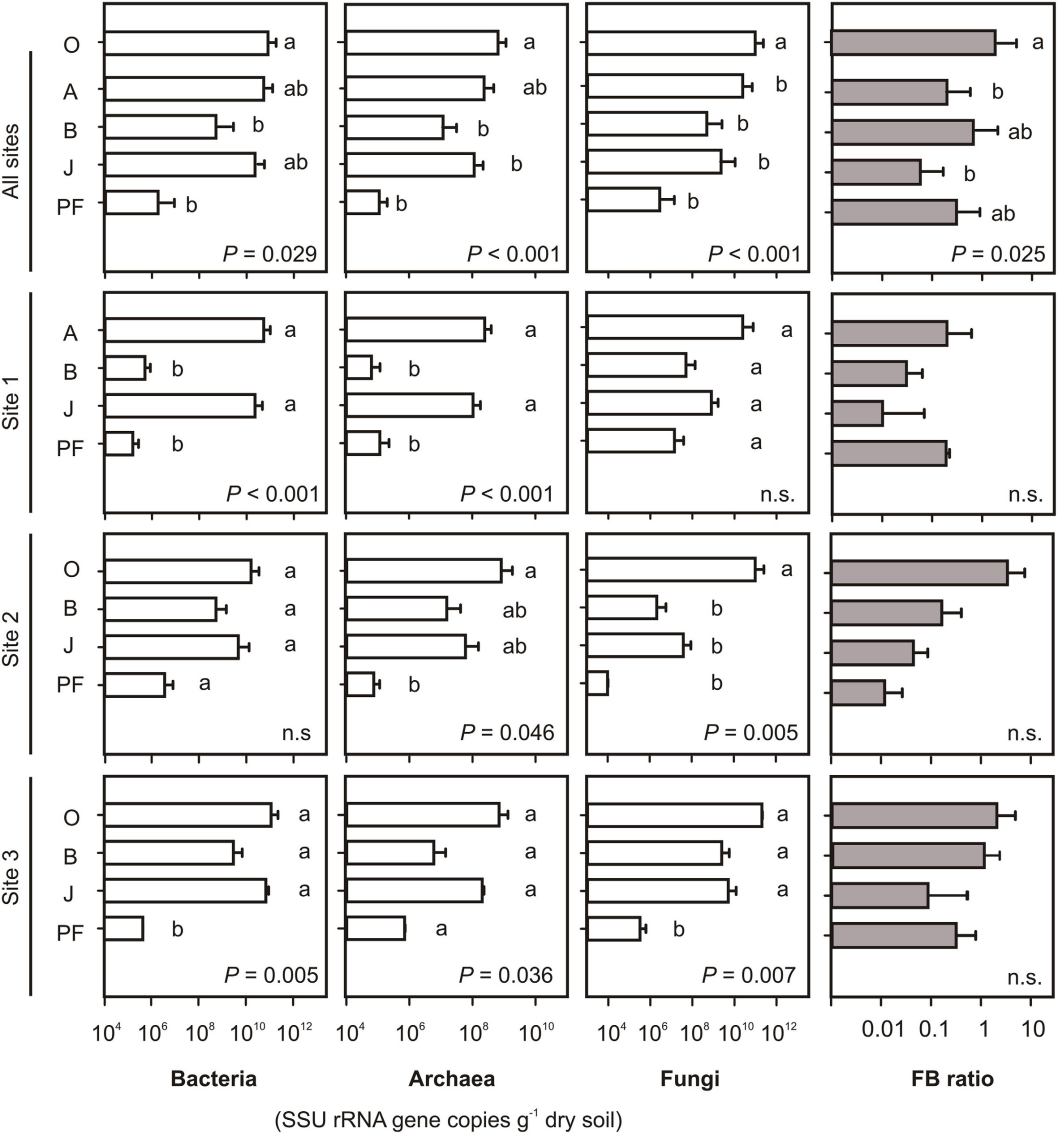
**Abundances of *Bacteria*, *Archaea* and *Fungi* shown as bacterial, archaeal and fungal SSU rRNA gene copy numbers per gram dry soil and fungal-bacterial (FB) ratios**. Note the difference in scaling of bacterial and fungal vs. archaeal abundances. Error bars for individual sites represent SD from 2 to 5 samples per soil type. Small letters indicate significant differences between soil horizons as determined by One-Way ANOVA and Tukey's HSD test. *P*-values indicate overall significant differences. O, organic topsoil; A, mineral topsoil; B, mineral subsoil; J, buried topsoil; PF, permafrost layer.

On average, bacterial SSU rRNA gene abundances per gram dry soil were highest in topsoil samples and buried topsoils and were two to four orders of magnitude lower in mineral subsoils and permafrost samples (Figure 1). In contrast, average fungal SSU rRNA gene copy numbers were one to two orders of magnitude higher in topsoils than in buried topsoils. Mineral subsoils did not show any significant differences in fungal SSU rRNA gene abundances when compared to buried topsoils, while permafrost samples exhibited the lowest fungal abundances. Thus, the disproportion in bacterial and fungal abundances in topsoils and buried topsoils led to highest fungal-bacterial (FB) ratios in O horizons (*FB* = 3.23, Figure 1), and lowest in J horizons (*FB* = 0.05). Significant differences in FB ratio were found between O and A topsoil horizons as well as between O and J horizons (One-Way ANOVA, *P* < 0.05). Archaeal gene copies followed the bacterial distribution pattern and were highest in topsoil horizons and buried topsoils. They were two to three orders of magnitude lower in mineral subsoils and permafrost samples. The fraction of archaeal SSU rRNA genes proportionally increased with depth being lowest in O and A horizons (0.01% of the total number of prokaryotic SSU rRNA gene copies) and highest in permafrost samples (0.4%).

General observations as described above were also apparent for the individual sampling sites, including highest bacterial, archaeal and fungal abundances in topsoils and buried topsoils, and lower abundances in subsoils and permafrost samples (Figure 1). While differences in bacterial and archaeal SSU rRNA gene abundances were significant at sites 1 and 3, differences at site 2 were only minor or not significant (Figure 1). Fungal SSU rRNA abundances at site 2 were significantly higher in topsoils than in any other horizon. At site 3, fungal abundance was significantly lower in permafrost samples than in topsoil and subsoil horizons as well as buried topsoils. Differences in FB ratios between horizons were not significant (Figure 1).

Similar patterns in bacterial, archaeal and fungal SSU rRNA gene distributions were found when soil organic carbon (OC) was taken into account (Figure S3). However, differences in gene abundances per gram OC between soil horizons were less pronounced when compared to calculations on the dry weight basis (represented by lower *P*-values or insignificant differences, Figure S3).

### Differences in prokaryotic community composition and diversity between sites and horizons

**Figure 2:**
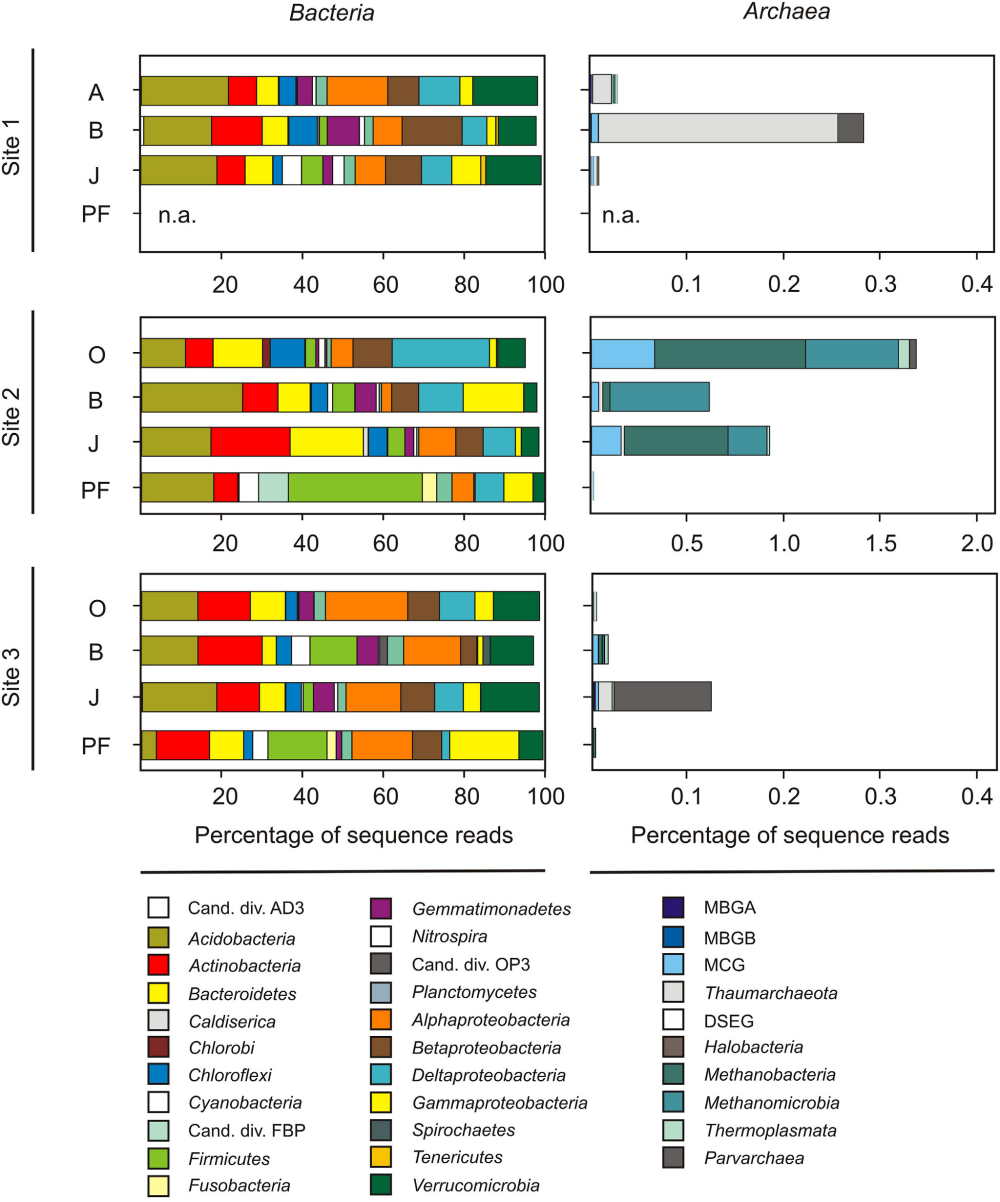
**Community structure as revealed from Illumina tag sequencing of the prokaryotic V4 region of the SSU rRNA gene. Left panels**: Percentage of OTUs affiliated to bacterial phyla, candidate divisions and proteobacterial classes (only shown if they represented at least 1% of all sequences in any of the samples). **Right panels**: Percentage of archaeal phyla, candidate divisions and classes. Cand. div., candidate division; MBGA, marine benthic group A; MBGB, marine benthic group B; MCG, miscellaneous crenarchaeotal group; DSEG, deep sea euryarchaeotal group; O, organic topsoil; A, mineral topsoil; B, mineral subsoil; J, buried topsoil; PF, permafrost layer. Note the difference in scaling of archaeal reads for site 2. (More detailed information the relative abundance of bacterial and archaeal classes in Table S3).

Twenty-seven samples covering the four different soil horizons (O and A, B, J, and PF) were subjected to Illumina tag sequencing of the V4 SSU rRNA gene region (sequence and metadata available from http://www.microbio.me/emp/, study no. 1034). Sequencing was successful for 20 of these samples (Table 3, Table S3) and yielded a total of 1,975,888 bacterial and archaeal SSU rRNA gene sequences after extensive read-quality filtering (see Materials and Methods for details) with 86% being taxonomically classified using closed-reference OTU picking. Bacterial and archaeal sequences clustered in 10,309 OTUs, representing 157 classes (147 bacterial and 10 archaeal) within 55 phyla (including 30 candidate phyla). The dominant phyla were *Proteobacteria* (α, β, δ, and γ), *Acidobacteria*, *Actinobacteria*, *Verrucomicrobia*, *Bacteroidetes*, and *Firmicutes* accounting for ~84% of all sequences (Figure 2). In addition, *Chloroflexi*, *Gemmatimonadetes*, *Planctomycetes, Cyanobacteria*, and *Nitrospirae* were present in all soil horizons, but at lower abundances (~12% of all sequences), and 38 other rare phyla (<0.5% each) were identified. Rare phyla included archaeal taxa that represented 0.3% of all sequence reads. The majority of archaeal reads was assigned to the classes *Methanomicrobia* and *Methanobacteria* within the *Euryarchaeota* comprising 39.8% of all archaeal reads. For site 2, sequence reads assigned to archaeal taxa comprised 0.9% of all reads obtained from site 2 samples, whereas they only accounted for 0.05 and 0.01% at sites 1 and 3, respectively (Figure 2). This finding was in line with lower archaeal SSU rRNA gene abundances at sites 1 and 3 than at site 2 (as determined by qPCR). Furthermore, the majority of sequences retrieved from site 2 was affiliated with methanogenic taxa of the families *Methanosarcinaceae*, *Methanobacteriaceae*, and *Methanosaetaceae* in the phylum *Euryarchaoeta*, whereas members of the *Thaumarchaeota* and several other non-methanogenic archaea dominated at sites 1 and 3 (Figure 2).

**Table 3:** **Sequencing statistics, diversity and evenness estimates^a^**.

**Site**	**Soil group**	**Number of samples**	**Number of sequences**	**Classified (%)**	**Number of OTUs**	**Chao1**	**Shannon**	**Inverse simpson**	**Pielou's evenness**
1	A	3	297648	85.0	3262±231 (a)	4209±291 (a)	6.36±0.11 (a)	175.4±41.2 (a)	0.79±0.01 (a)
	B	2	113553	87.1	1402±469 (a)	2095±392 (a)	5.78±0.19 (a)	129.4±11.8 (a)	0.80±0.02 (a)
	J	2	477139	85.6	2591±3335 (a)	3394±4035 (a)	4.93±1.98 (a)	89.5±91.6 (a)	0.70±0.07 (a)
	PF	0	ns	ns	ns	ns	ns	ns	ns
2	O	1	113205	80.3	3126 (a)	4133 (a)	6.18 (a)	138.4 (a)	0.77 (a)
	B	2	185477	87.9	851±336 (b)	1422±233 (b)	4.12±0.45 (ab)	20.4±11.2 (b)	0.61±0.03 (a)
	J	3	272337	86.7	1131±282 (b)	1780±479 (b)	4.62±0.33 (ab)	35.0±13.0 (b)	0.66±0.07 (a)
	PF	1	35414	91.1	274 (b)	530 (b)	3.49 (b)	22.6 (b)	0.62 (a)
3	O	2	207447	85.3	3803±240 (a)	4928±321 (a)	6.64±0.03 (a)	250.0±16.8 (a)	0.81±0.01(a)
	B	1	59861	88.3	483 (a)	877 (a)	4.38 (a)	51.8 (a)	0.71 (b)
	J	2	169150	85.0	2391±1755 (a)	3300±2004 (a)	6.10±0.57 (a)	166.4±69.2 (a)	0.80±0.01 (a)
	PF	1	44657	89.6	619 (a)	1203 (a)	4.48 (a)	36.1 (a)	0.70 (b)

a *Means and standard deviations were calculated from replicate soil samples classified into the respective soil group at the respective site. Detailed data for each soil sample can be found in Table S3 (Supplementary Material). Small letters in brackets indicate significant differences as determined by One-Way ANOVA and Tukey's HSD test*.

*Ns, sequencing not successful*.

**Figure 3:**
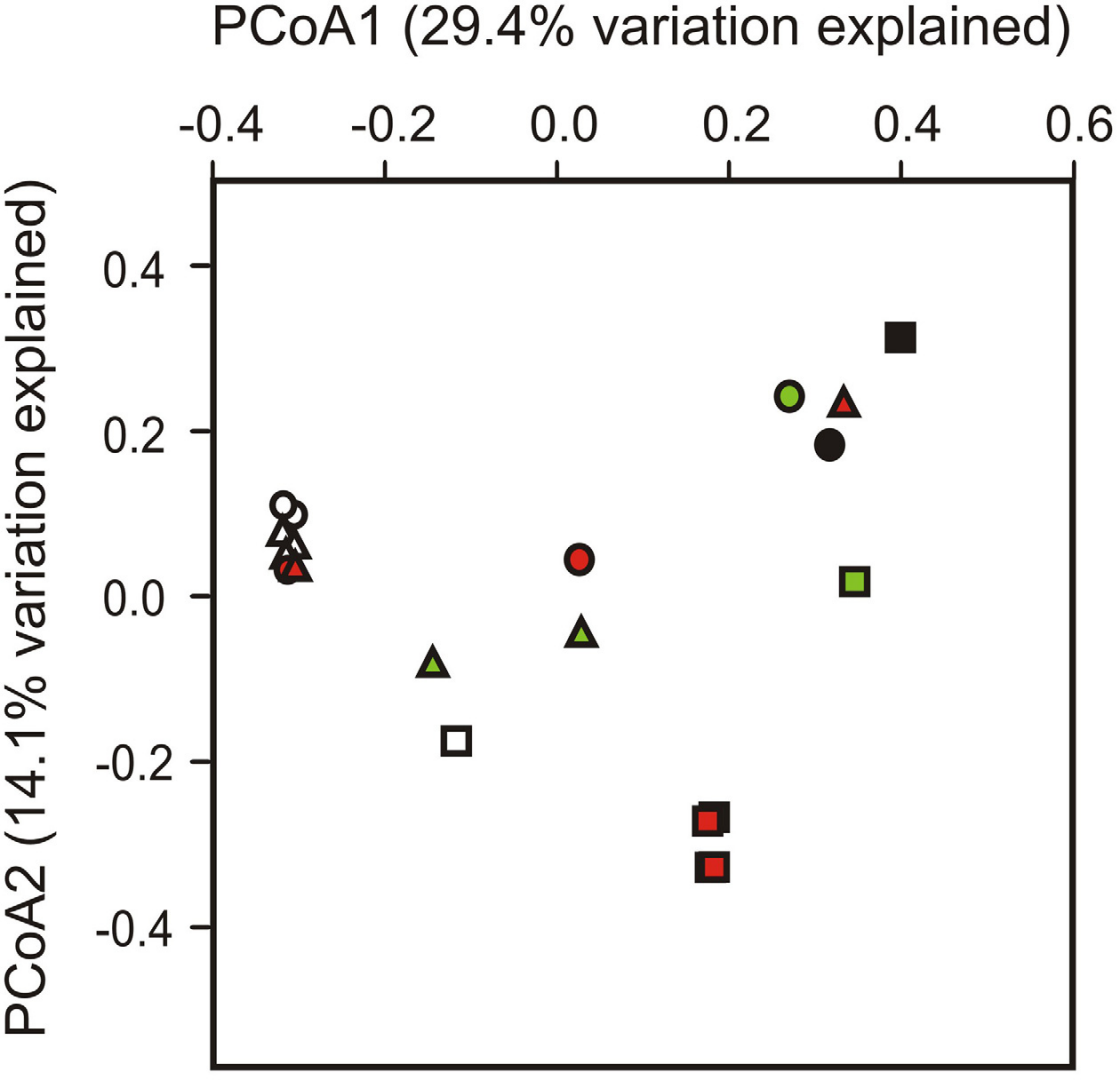
**Phylogenetic dissimilarity between individual soil samples as assessed by principal coordinate analysis (PCoA) based on unweighted UniFrac distances**. Sites are represented by symbols (site 1, triangles; site 2, squares; site 3, circles), soil horizons are color-coded (O and A, topsoils: white; B, subsoils: green; J, buried topsoils: red; PF, permafrost layer: black).

To test whether different sites and/or soil horizons harbor distinct communities, we applied PERMANOVA and PCoA. A Bray-Curtis distance matrix of square-root transformed, relative OTU abundances was analyzed by PERMANOVA in a three-factor design to test for differences between sampling sites, soil horizons and replicate soil pits. Significant differences were returned for sampling site (Pseudo-*F* = 10.27, *p*(perm) = 0.001) and soil horizon (Pseudo-*F* = 6.12, *p*(perm) = 0.007), but not for the soil pit factor (Pseudo-*F* = 2.42, *p*(perm) = 0.097). No interactions between factors (site, horizon, pit) were apparent (*P* > 0.05 in all instances). PCoA based on unweighted UniFrac distances was used to visualize clusters of similar communities according to sampling site, soil horizon and soil pit (Figure 3). Most significant differences were found between sampling sites (Pseudo-*F* = 29.96, *p*(perm) = 0.001), followed by soil horizons (Pseudo-*F* = 22.03, *p*(perm) = 0.002), and soil pits (Pseudo-*F* = 5.93, *p*(perm) = 0.025). Topsoil samples from sites 1 and 3 formed a distinct cluster that also included two out of four of the buried soil samples from these sites (samples A4 and G4), but excluded topsoil and buried topsoil samples from site 2 (Figure 3). In contrast, buried topsoils from site 2 formed a distinct cluster and were thus separated from the corresponding topsoil sample as well as from samples from sites 1 and 3 (Figure 3). Subsoil and permafrost samples did not show consistent clusters according to sampling site and/or soil horizon.

Species richness and diversity were generally higher in topsoils than in buried topsoils, mineral subsoil horizons and the underlying permafrost (Table 3). However, this difference was only significant at site 2 (One-Way ANOVA, *P* < 0.05). Samples from buried topsoils at sites 1 and 3 were highly variable in species richness and diversity resulting in statistically insignificant differences between horizons (Table 3).

### Potential enzyme activities and correlations with microbial community structure

**Figure 4:**
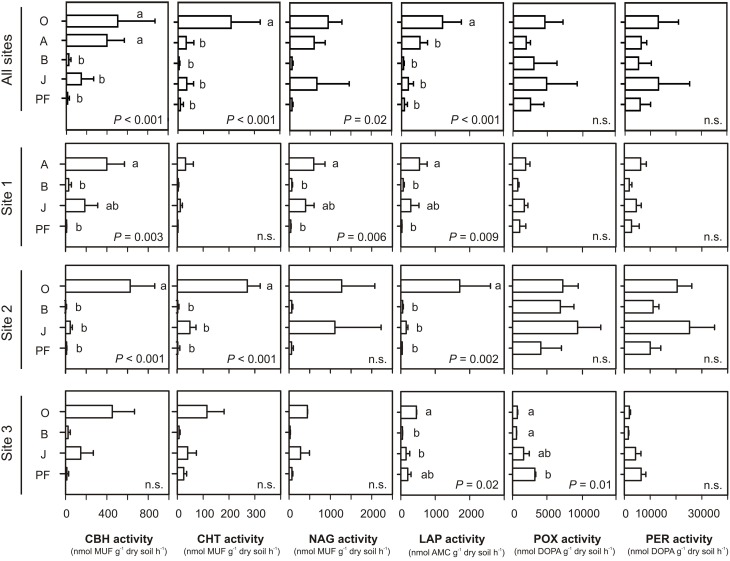
**Potential extracellular enzymes activities calculated per gram dry soil**. Means and standard deviations were calculated from soil samples classified into each soil group. Small letters indicate significant differences between soil horizons as determined by One-Way ANOVA and Tukey's HSD test. CBH, 1,4-β-cellobiohydrolase; CHT, 1,4-β-poly-N-acetylglucosaminidase; NAG, β-N-acetylglucosaminidase; LAP, leucine aminopeptidase; POX, phenol oxidase; PER, peroxidase; MUF, 4-methylumbelliferyl; AMC, aminomethylcoumarin; DOPA, L-3,4-dihydroxyphenylalanin; O, organic topsoil; A, mineral topsoil; B, mineral subsoil; J, buried topsoil; PF, permafrost layer. For NAG activity, an overall significant *P*-values was obtained, but no siginifcant pairwise-differences between horizons were found (lowest pairwise *P*-values for O vs. B: 0.058, and O vs. PF: 0.067).

Potential hydrolytic enzyme activities (per gram dry soil) were highest in topsoil samples (O and/or A), followed by buried topsoils, mineral subsoils and permafrost samples (Figure 4). This difference was significant for CBH, CHT, LAP, and to some extent for NAG (One-Way ANOVA, *P* < 0.05). Potential NAG activity was similarly high in topsoil and buried topsoil samples and significantly lower in mineral subsoils and permafrost samples. Potential oxidative enzyme activities were highest in samples from topsoils (O) and buried topsoils (Figure 4). However, due to the high variability between sampling sites, differences in POX and PER activities in topsoils, mineral subsoils and permafrost samples were not significant (One-Way ANOVA, *P* > 0.05). Potential POX and PER activities were significantly higher in active layer samples from site 2 compared to the corresponding soil samples from sites 1 and 3 (One-Way ANOVA, *P* > 0.05, Figure 4, Table S4). In contrast, only few significant differences were found for the potential hydrolytic enzyme activities in samples from the same soil horizon at the different sites (Figure 4, Table S4).

When potential enzyme activities were calculated per gram organic carbon (OC), differences between horizons became smaller and less significant for hydrolytic enzymes Figure S4). For oxidative enzymes, the pattern changed to mineral subsoils (and permafrost samples) exhibiting a higher potential activity on a per gram OC basis than topsoils and buried topsoils. Differences for same soil horizons at the different sites got less pronounced for oxidative enzyme activities and did not show a clear pattern for hydrolytic enzymes (Table S5).

**Figure 5:**
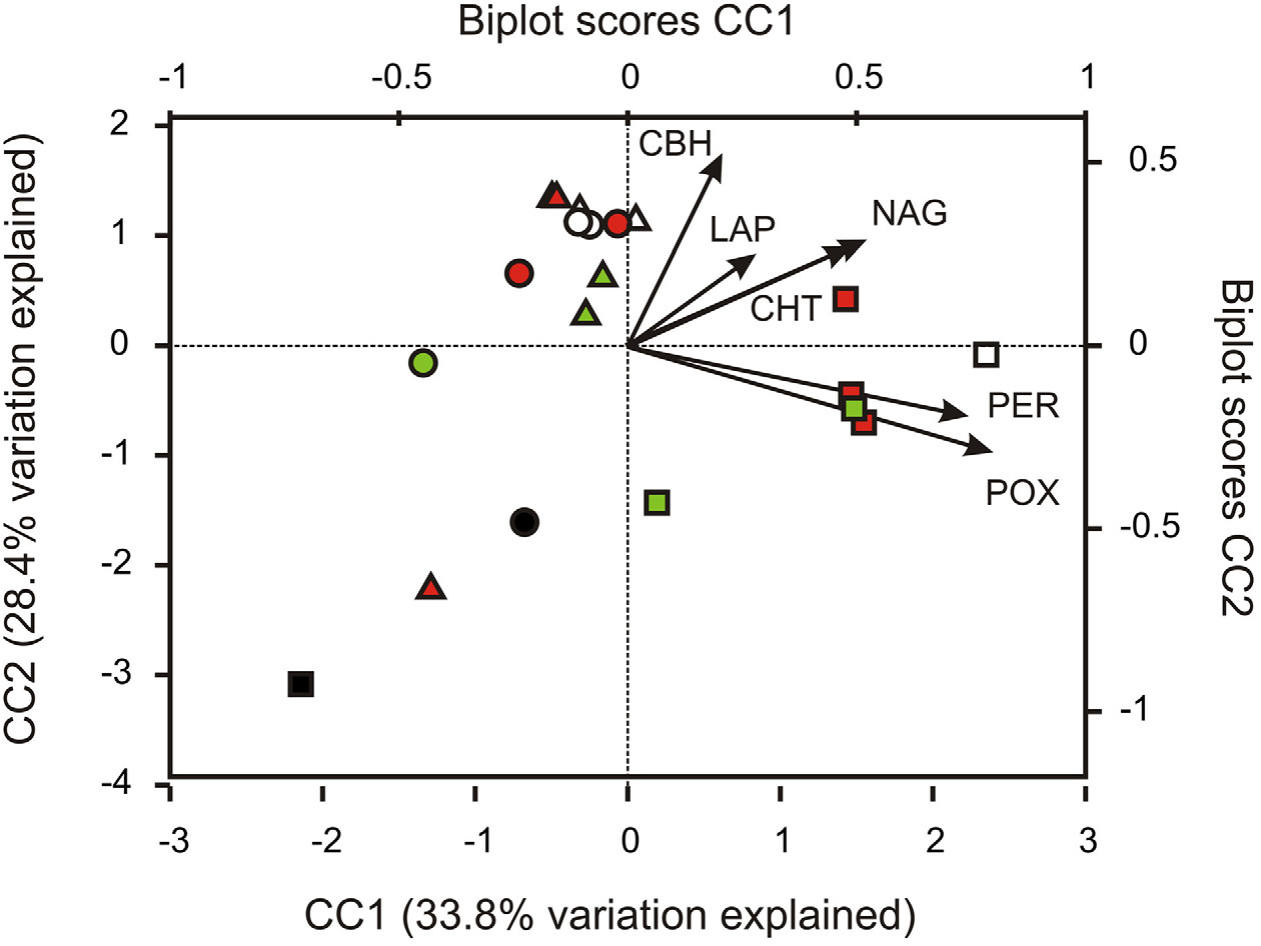
**Canonical correspondence analysis (CCA) of potential enzymatic activities' interactions with changes of the relative abundance of taxonomically classified OTUs**. CBH, 1,4-β-cellobiohydrolase; CHT, 1,4-β-poly-N-acetylglucosaminidase; NAG, β-N-acetylglucosaminidase; LAP, leucine aminopeptidase; POX, phenol oxidase; PER, peroxidase. Sites are represented by symbols (site 1, triangles; site 2, squares; site 3, circles), soil horizons are color-coded (O and A, topsoils: white; B, subsoils: green; J, buried topsoils: red; PF, permafrost layer: black).

CCA was used to examine whether potential enzyme activities were correlated to changes in community structure (Figure 5). The first three axes of the model explained 77% of the total variation within the OTU abundance data in relation to the six enzyme activities measured. Samples from site 2 were separated from samples from sites 1 and 3 along the first axis (CC1). POX and PER activities exerted the strongest positive influence on axis 1. It was furthermore indicated that the spatial distribution of several classes within the *Actinobacteria* (e.g., class *Actinobacteria*) and the *Bacteroidetes* (e.g., class *Bacteroidia*), as well as putatively anaerobic members of the *Firmicutes* (*Clostridia*), the *Chloroflexi* (*Anaerolinea*, *Dehaloccoidetes*), and methanogenic *Euryarchaeota* (*Methanomicrobia*) were positively correlated with potential POX and PER activities (Figure S5).

### Distribution of selected taxa associated with known functions

**Figure 6:**
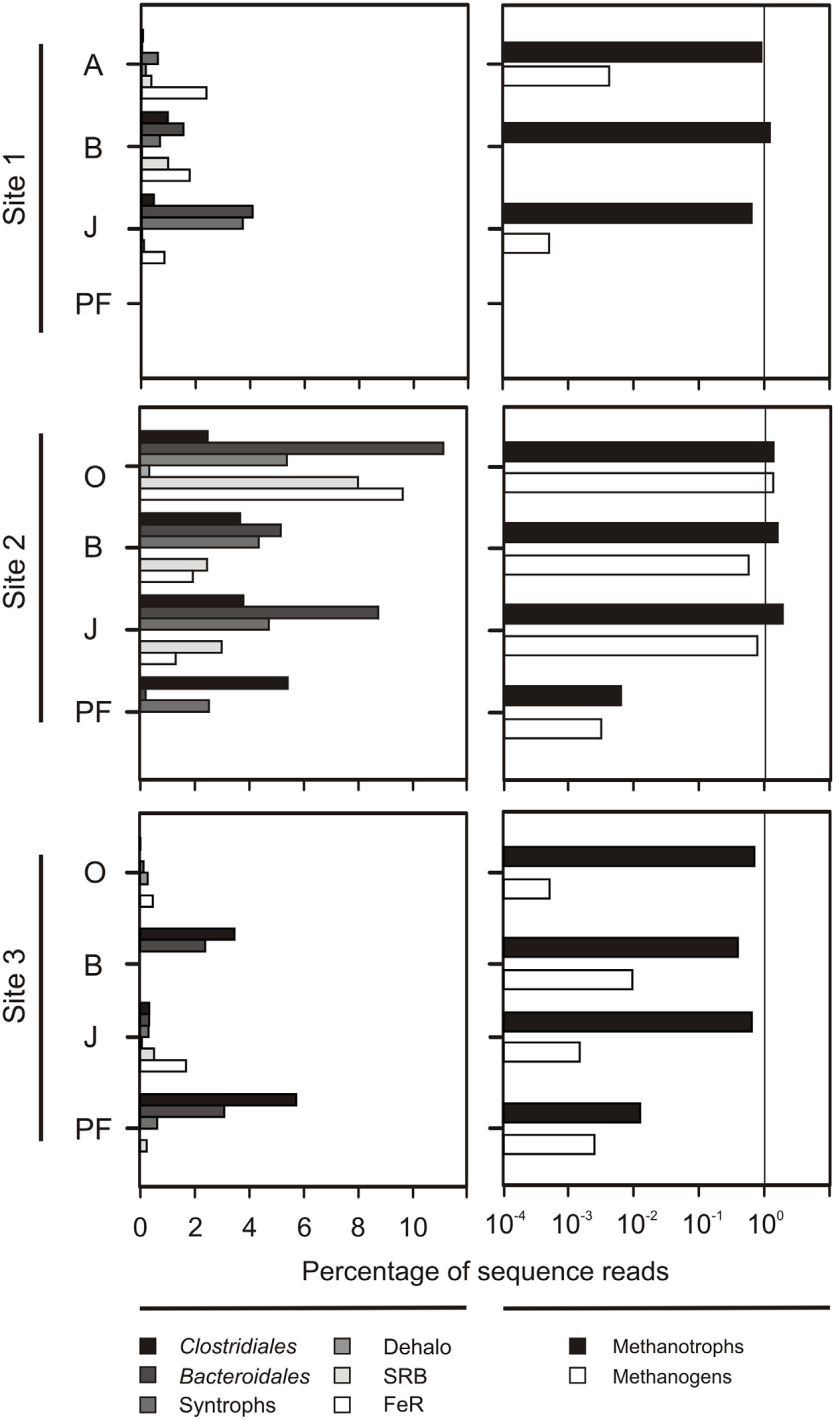
**Relative abundance of presumably anaerobic taxa (left panels) and taxa putatively involved in methane cycling (right panels). Left panels**: Presumably anaerobic taxa including strict fermenters (*Clostridiales*, *Bacteroidales*), syntrophs (*Syntrophomonadaceae*, *Syntrophaceae*, *Syntrophobacteraceae*), dehalo respirers (Dehalo; Anaeromyxobacter), sulfate reducers (SRB; *Desulfobacteraceae*, *Desulfobulbaceae*, *Desulfohalobiaceae*, *Desulfovibrionaceae*), and metal reducers (FeR; *Desulfuromonadaceae*, *Geobacteraceae*, *Pelobacteraceae*). **Right panels**: Methanogenic archaea *(Methanobacteria, Methanomicrobia, and Thermoplasmata* affiliated with *Methanomassiliicoccaceae)* and methanotrophic bacteria (MOB I + II types, and *Verrucomicrobia affilated with Methylacidiphilae*).

Sequences matching putative anaerobic taxa (e.g., fermenters, sulfate reducers, metal reducers, methanogens) were highly abundant in the wet fen site accounting for up to 38% of all sequences in the O horizon (Figure 6). While the relative contribution of these taxa to the total community was similarly high in all horizons at site 2 (except for the permafrost sample), their proportion increased with depth at sites 1 and 3 (Figure 6). Here, there relative abundance increased from 2.32% in the O horizons to 9.8% in the permafrost (averaged over all samples from the respective horizons at these sites). Methanogenic archaea were particularly abundant at the wet fen site (0.5–1.3% in the active layer horizons). A high diversity of aerobic methane-oxidizing bacteria (MOB) was found at all sites and in all horizons and included MOB of type I (*Methylococcaceae*) and type II (*Methylobacteriaceae*, *Methylocystaceae*, *Beijerinckaceae*), and the recently described *Methylacidiphilae* within the phylum *Verrucomicrobia* (Table S3). Type I MOB were more abundant than type II MOB in mineral soil horizons in the active layer (38 vs. 18% of all MOB, respectively). Type II MOB predominated in topsoils and buried topsoils (38 and 46% of all MOB, respectively) together with a large fraction of putative methanotrophic *Verrucomicrobia* (52 and 40% of all MOB, respectively).

## Discussion

The top permafrost in northeastern Greenland is thawing at present, making stored carbon prone to microbial decomposition and potentially leading to an increased future release of CO_2_ and CH_4_ (Elberling et al., 2013). However, the knowledge on microbial communities in permafrost-affected soils in Greenland is rather scarce, restricted to only few sampling sites (Ganzert et al., 2014) and limited to certain physiological or functional guilds (such as methanotrophs) (Bárcena et al., 2011). Thus, our study provides valuable information on the structure, diversity and SOM degradation potential of microbial communities in permafrost-affected soils, and thereby contributes to the understanding of the impact of climate warming on microorganisms and *vice versa* (Graham et al., 2012; Jansson and Tas, 2014).

Our findings support recent studies in the Siberian Arctic that described microbial communities in topsoils buried by cryoturbation as distinct from unburied topsoil communities (Gittel et al., 2014; Schnecker et al., 2014). Fungal-bacterial ratios differed significantly between organic (O) and mineral (A) topsoil horizons as well as between topsoils (O) and buried topsoils (J), being lowest in the latter. This corroborated the hypothesis that fungi play a critical role in the delayed decomposition of SOM in buried topsoils (Gittel et al., 2014; Schnecker et al., 2014). However, in contrast to the strong effect of soil horizon on the beta diversity in permafrost-affected soils from Siberia (Gittel et al., 2014), buried topsoils from Greenland were more heterogeneous both in diversity, abundance and enzymatic activities. For instance, the bacterial core community (OTUs present in all buried topsoil samples) consisted of only twenty-one OTUs comprising members of the phyla *Acidobacteria*, *Actinobacteria*, *Bacteroidetes*, *Chloroflexi*, *Firmicutes*, and the *α-, β-, δ-Proteobacteria*. This core community accounted for 4.5, 15.0, and 3.4% of the total community at sites 1, 2, and 3, respectively. The most abundant OTU (Greengenes OTU ID 637267) in buried topsoils was affiliated with the family *Intrasporangiaceae* (*Actinobacteria*). Its abundance was particularly high in buried topsoils from site 2 (8.3% of the total community). Although this OTU was also present in 90% of all other soil samples, it only accounted for an average of 1.7% to the total community in non-buried topsoils, mineral subsoils and permafrost samples. Members of the *Intrasporangiaceae* also constituted a major fraction of the prokaryotic community in mineral subsoils and buried topsoils from the Siberian Arctic, where they accounted for up to 30 and 47% of the total community, respectively (Gittel et al., 2014). It has been hypothesized that members of this family have adapted to the low availability of carbon and energy sources and the harsh abiotic conditions in permafrost-affected soils and cryoenvironments explaining their ubiquitous distribution in these habitats (Hansen et al., 2007; Steven et al., 2008; Yergeau et al., 2010; Yang et al., 2012). Recent studies suggested that they are able to degrade cellulose, cellobiose (which is a product of enzymatic cellulose hydrolysis), and lignin in agricultural soils (Schellenberger et al., 2010; Giongo et al., 2013). Indeed, CCA analysis indicated a linkage between the distribution of the class *Actinobacteria* and the potential activity of peroxidases and phenol oxidases (Figure S5). This supports the hypothesis that *Actinobacteria* might significantly contribute to lignin degradation and transformation in periodically anoxic environments (e.g., water-logged soil horizons), where fungal degradation activities are restricted (Boer et al., 2005; DeAngelis et al., 2011; Gittel et al., 2014). Growing molecular evidence furthermore points to the ecological importance of bacterial laccases, the probably largest class of phenol oxidases, as they were found to be highly diverse and present in a wide range of bacterial phyla, amongst others including *Actinobacteria*, *Acidobacteria, Bacteroidetes*, *Firmicutes*, *Proteobacteria*, and *Verrucomicrobia*, as well as in archaea (Nakamura et al., 2003; Ausec et al., 2011a,b; Freedman and Zak, 2014). It has been argued that bacterial laccase expression might be more efficient compared to fungal laccases due to the lack of introns and posttranslational modifications (Ausec et al., 2011b) and that both their diversity and activity in soils might be much greater than that of their fungal equivalent (Kellner et al., 2008). Potential oxidative enzyme activities (per gram dry soil) did not show significant differences between soil horizons and thus did not follow the change in community composition, and the decrease in fungal and bacterial abundances and total microbial biomass with depth (Figure 4). This might either suggest that the abundance, and by extension the activity, of fungi producing these enzymes did not change with depth, and that the decrease in total fungal abundance resulted from a decreased abundance of saprothrophic fungi. The latter have previously been shown to be correlated to the activity of hydrolytic enzymes and thus linked to soil properties such as C, N, and moisture (Talbot et al., 2013). It might however also indicate that bacteria of the above mentioned phyla resume and more efficiently express oxidative enzyme activities, while ectomycorrhizal fungi decreased with depth as a result of lacking plant support, e.g., root exudates (Talbot et al., 2008; Gittel et al., 2014). Furthermore and in contrast to hydrolytic enzymes, both phenol oxidases and peroxidase unspecifically catalyze reactions that do not necessarily lead to the acquisition of carbon and nutrients, e.g., mitigation of toxicity of phenols and metals, or antimicrobial defense (Sinsabaugh, 2010). Thus, soil chemistry (i.e., organic matter content) had less of an effect on their activity resulting in insignificant differences between soil horizons (Figure 4).

We found a pronounced effect of the sampling site and concomitant abiotic factors on community composition (Figures 2, 3, Figure S2), with the wet fen site (site 2) being a putative hot spot for anaerobic degradation processes such as methanogenesis and fermentation (Figure 6). A large variety of presumably anaerobic taxa including strict fermenters, syntrophs, sulfate reducers, and metal reducers showed particularly high relative abundances in the active layer of the wet fen site presumably favored by high moisture and low oxygen levels (Figure 6). In accordance with a recent metagenomic study on arctic peat soil (Lipson et al., 2013), putative members of these functional guilds were also detected in permafrost samples suggesting that the respective processes occur both in the active layer and the underlying permafrost. With increasing temperatures in the Northern Latitudes and deepening of the active layer, higher soil moisture and lower oxygen levels will become more prevalent leading to shifts in microbial community composition and functionality toward anaerobic degradation processes (Allan et al., 2014; Frank-Fahle et al., 2014).

Net methane release from arctic soils is regulated by the balance between methane production by anaerobic methanogenic archaea and its conversion to CO_2_ by primarily aerobic methane-oxidizing bacteria (Liebner et al., 2009; Graef et al., 2011; Allan et al., 2014). Both activities are affected by a variety of environmental factors such as temperature, water table depth, substrate availability and redox potential, and their balance will eventually determine whether arctic soils act as a source or a sink for methane. Both hydrogenotrophic and acetoclastic methanogens were found in the active layer of all our sampling sites, but were particularly abundant at the wet fen site (Figure 6). Significantly higher moisture and thus lower oxygen levels in the active layer of site 2 probably provided more favorable conditions for methanogens than at sites 1 and 3. Members of the *Methanobacteriales* and the *Methanomicrobiales* (using H_2_/CO_2_ for methanogenesis) dominated in the topsoil horizons, whereas *Methanosarcinales* dominated in mineral subsoils. The latter are able to generate methane from a broad range of substrates including acetate, H_2_/CO_2_ and methylated compounds (e.g., methanol). This depth-related succession from a hydrogenotrophic to a metabolically more flexible methanogenic community has been found in other permafrost environments, such as arctic wetlands and peat soils, and has been attributed to advantages in the competition for substrates and their independence from syntrophic interactions (Høj et al., 2005; Tveit et al., 2012; Lipson et al., 2013; Frank-Fahle et al., 2014).

In line with previous reports from various cold environments (Liebner et al., 2009; Yergeau et al., 2010; Tveit et al., 2012), type I MOB outnumbered type II MOB in mineral soil horizons in the active layer, while type II MOB predominated in topsoils and buried topsoils together with a large fraction of putative methanotrophic *Verrucomicrobia* (52 and 40% of all MOB, respectively). Together with few other studies (Barbier et al., 2012; Martineau et al., 2014), our results thus indicated that MOB diversity was much larger than expected and—depending on the soil horizon and its properties—is clearly not restricted to type I MOB. However, the environmental factors (pH, CH_4_ partial pressure, soil temperature) determining the dominance of one or the other MOB type, the balance between methane oxidation and methanogenesis and the ecological implications for arctic environments need further research.

The collective results presented here shed light on the highly diverse microbial communities in permafrost-affected soils in Northeast Greenland, their enzymatic degradation potential and the distribution of functional guilds involved in the anaerobic degradation of SOM (fermentation, methanogenesis). Site-specific differences were apparent between the typical tundra sites and the wet fen site, the latter being a potential hot spot for degradation activities. In addition, shifts in community composition between unburied and buried topsoils (decrease in fungal abundance and a predominance of *Actinobacteria* and other potential bacterial laccase producers) and stable oxidative enzyme activities with depth supported the hypothesis that bacteria might resume the role of fungi in the degradation of phenolic compounds such as lignin.

## Author contributions

Antje Gittel, Jiří Bárta, Tim Urich and Andreas Richter designed the research presented in this manuscript. Antje Gittel directed and performed the molecular work and sequence data analyses on prokaryotic communities. Jiří Bárta and Iva Kohoutová performed the quantification of fungal communities. Petr Čapek contributed with data on microbial biomass. Jörg Schnecker and Birgit Wild determined enzyme activity potentials and soil geochemical properties. Samples were collected by Christina Kaiser and Andreas Richter. Antje Gittel performed all statistical analyses. Antje Gittel, Jiří Bárta and Tim Urich wrote the manuscript with input from Christina Kaiser, Jörg Schnecker, Birgit Wild, Vigdis L. Torsvik, and Andreas Richter.

### Conflict of interest statement

The authors declare that the research was conducted in the absence of any commercial or financial relationships that could be construed as a potential conflict of interest.
