# Supplementary material for: Site- and horizon-specific patterns of microbial community structure and enzyme activities in permafrost-affected soils of Greenland
Source: Front Microbiol. 2014 Oct 16;5:541. doi: 10.3389/fmicb.2014.00541 (PMC4199454; doi:10.3389/fmicb.2014.00541)
Supplement: Supplementary file 1 [file DataSheet1.PDF]

Supplementary Material

Figure S1. Sampling sites, dominant vegetation and sampling scheme.

Figure S2. PCA ordination plot resulting from soil properties (moisture, TOC. TN, CN ratio) and microbial biomass (Cmic, Nmic). Data were standardized to zero mean and standard deviation. Sites are represented by symbols (site 1: triangles, site 2: squares, site 3: circles), soil horizons are color-coded (O and A, topsoils: white; B, subsoils: green; J, buried topsoils: red; PF, permafrost layer: black).

Figure S3. Abundances of *Bacteria*, *Archaea* and *Fungi* shown as bacterial, archaeal and fungal SSU rRNA gene copy numbers per gram organic carbon (OC) (logarithmic scales). Note the difference in scaling of bacterial and fungal *versus* archaeal abundances. Error bars for individual sites represent SD from 2 to 5 samples per soil type. Small letters indicate significant differences between soil horizons as determined by one-way ANOVA and Tukey's HSD test (log-transformed data). *P* values indicate overall significant differences. O: organic topsoil, A: mineral topsoil, B: mineral subsoil, J: buried topsoil, PF: permafrost layer.

Figure S4. Potential extracellular enzymes activities calculated per gram organic carbon (OC). Means and standard deviations were calculated from soil samples classified into each soil group. Small letters indicate significant differences between soil horizons as determined by one-way ANOVA and Tukey's HSD test (no data transformation). Note that CBH activites had to be log-transformed prior to analysis to meet the conditions of normality and homogeneity of variance. CBH: 1,4-β-cellobiohydrolase, CHT: 1,4-β-poly-N-acetylglucosaminidase, NAG: β-N-acetylglucosaminidase, LAP: leucine aminopeptidase, POX: phenoloxidase, PER: peroxidase. MUF: 4-methylumbelliferyl, AMC: aminomethylcoumarin, DOPA: L-3,4-dihydroxyphenylalanin. O: organic topsoil, A: mineral topsoil, B: mineral subsoil, J: buried topsoil, PF: permafrost layer.

Figure S5. Canonical correspondence analysis (CCA) ordination plots showing relationships between prokaryotic community structure (relative abundance of taxonomically classified OTUs), archaeal and bacterial classes (panels A and B, respectively) and potential enzymatic activities. Sites are represented by large symbols (site 1: triangles, site 2: squares, site 3:

circles), soil horizons are color-coded (O and A, topsoils: white; B, subsoils: green; J, buried topsoils: red; PF, permafrost layer: black). Small circles represent archaeal (magenta, panel A) and bacterial (grey, panel B) classes, respectively. For clarity, bacterial classes are only shown if they are affiliated with the most abundant phyla (for details refer to Figure 2). To enhance visibility, bacterial classes referred to in the manuscript (class within the *Actinobacteria* and the *Bacteriodetes*, as well as *Clostridia*, *Anaerolineae*, *Dehalococcoidetes*) are shown as yellow diamonds. CBH: 1,4-β-cellobiohydrolase, CHT: 1,4-β-poly-N-acetylglucosaminidase, NAG: β-N-acetylglucosaminidase, LAP: leucine aminopeptidase, POX: phenoloxidase, PER: peroxidase. MBGA: marine benthic group A, MBGB: marine benthic group A, MCG: miscellaneous crenarchaeotal group, DSEG: deep sea euryarchaeotal group.

Table S1. Sampling site description.

Table S2. Soil sample description and geochemical properties.

Table S3. Taxonomic distribution of bacterial and archaeal SSU rRNA amplicon reads (relative abundance, %).

Table S4. One-way ANOVA comparisons (*P* values) of extracellular enzyme activity potentials per gram dry soil in same soil horizons at the different sampling sites. P values < 0.05 are marked. CBH: 1,4-β-cellobiohydrolase, CHT: 1,4-β-poly-N-acetylglucosaminidase, NAG: β-N-acetylglucosaminidase, LAP: leucine aminopeptidase, POX: phenoloxidase, PER: peroxidase.

Table S5. One-way ANOVA comparisons (*P* values) of extracellular enzyme activity potentials per gram organic carbon (OC) in same soil horizons at the different sampling sites. P values < 0.05 are marked. CBH: 1,4-β-cellobiohydrolase, CHT: 1,4-β-poly-N-acetylglucosaminidase, NAG: β-N-acetylglucosaminidase, LAP: leucine aminopeptidase, POX: phenoloxidase, PER: peroxidase.

Figure S1

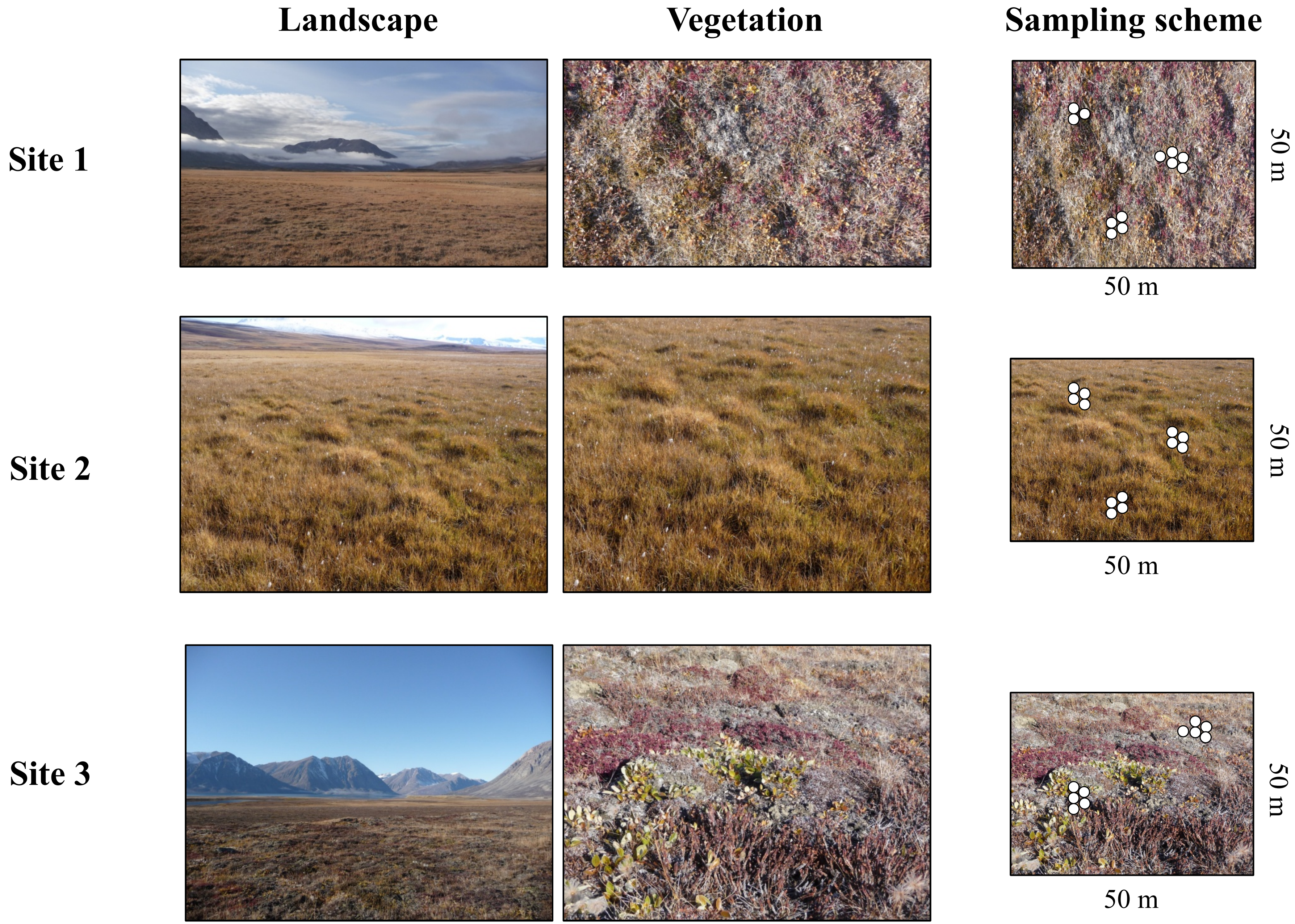
Landscape
Vegetation
Sampling scheme
Site 1
Site 2
Site 3
50 m
50 m
50 m
50 m
50 m
50 m

Figure S2
(Gittel et al. FiMB 2014)

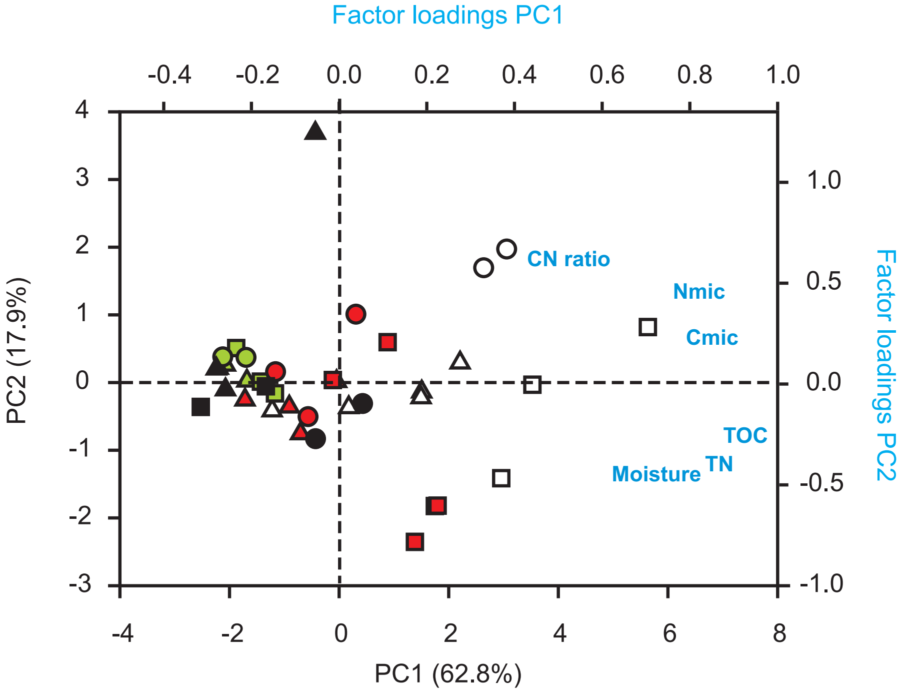

Figure S3
(Gittel et al. FiMB 2014)

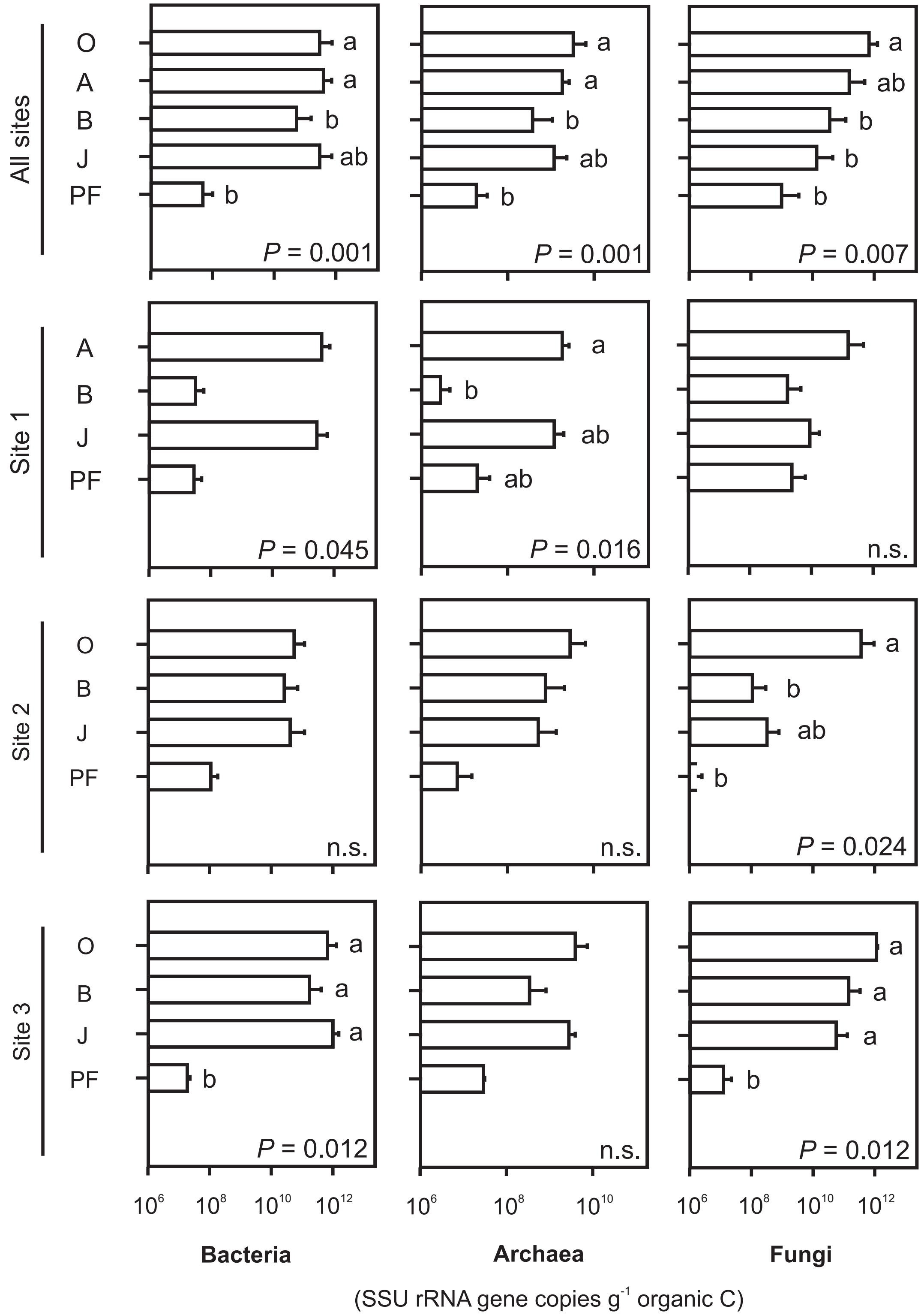

Figure S4
(Gittel et al. FiMB 2014)

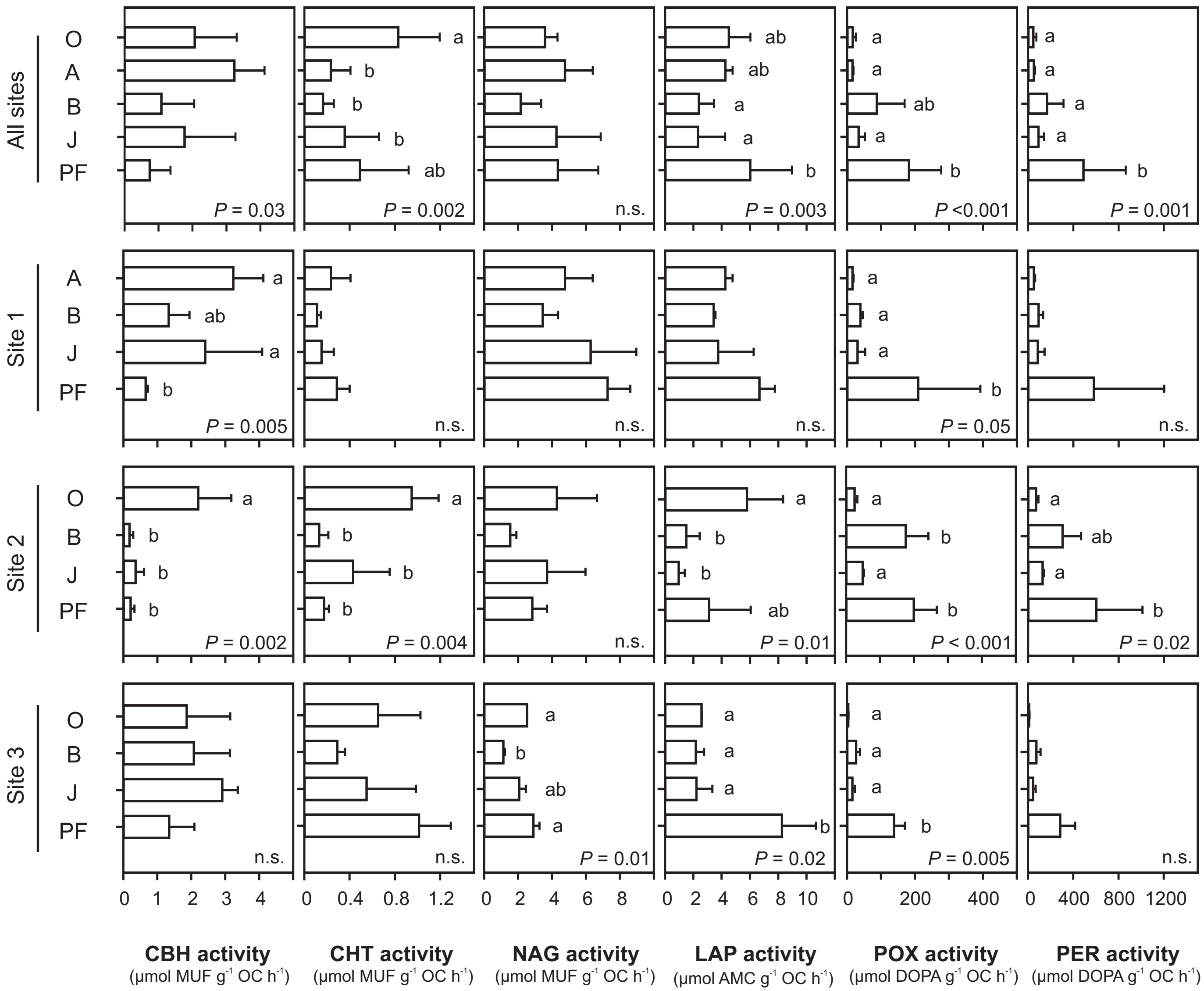

Figure S5
(Gittel et al. FiMB 2014)

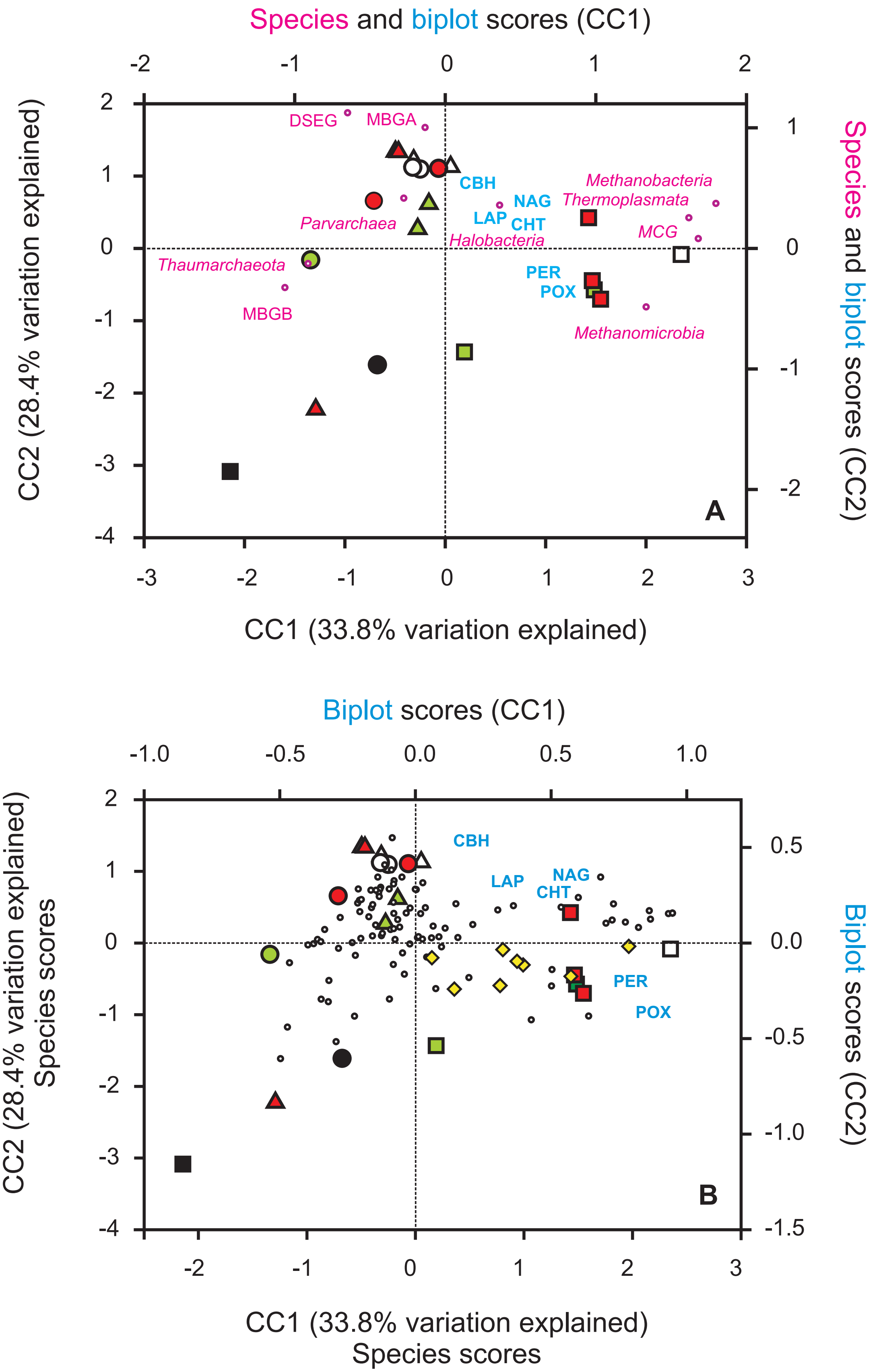

Table S1. Sampling site description.

Site	Site description	Vegetation	Replicate plots	Active layer depth (cm)
**1**	Tundra vegetation with old frost boils (already re-vegetated by lichens and some higher plants, e.g. *Dryas octopetala*) and some frost cracks	*Vaccinium uliginosum*, *Salix arctica*, *Carex* sp., *Polygonum viviparum*, *Dryas octopetala*, mosses, lichens on bare soil patches	A	48
			B	48
			C	55
**2**	Wet fen with ~ 5 % mossy hummocks on flat surface	*Carex* species, *Eriophorum angustifolium* (~5%), mosses, several grasses	D	42
			E	45
			F	55
**3**	Almost flat site with active frost boils and earth hummocks (differences in relief up to 30 cm)	*Cassiope tetragona*, *Salix arctica*, *Vaccinium uliginosum*, *Dryas octopetala*, grasses, *Carex* sp., mosses; some lichens on bare soil patches	G	46
			H	42

Table S2. Soil sample description and geochemical properties.

Plot	Sample	Soil horizon *	Soil group *	Depth (cm)	Special features	Moisture (%)	Total C (%)	Total N (%)
A	A1	O	A	0-20, partly down to 35		51.4	15.72	1.01
	A2	OA	A	20-35	Mainly A, some plant remains	41.5	10.38	0.75
	A3	B	B	43-48	Slightly gleyic	19.0	1.69	0.12
	A4	Ajj	J	35-40, and 43-48		36.3	7.64	0.51
	A5	Bff	PF	48-65		19.2	0.46	0.02
B	B1	OA	A	0-30		53.1	14.48	0.94
	B2	B	B	30-38	Slightly gleyic	26.3	2.97	0.22
	B4	Ajj	J	38-48	Hardly decomposed, coarse organic material	43.9	10.08	0.59
	B5	Ajj	J	38-55 (always below B4)	Well decomposed, fine black organic material	27.1	4.15	0.31
	B6	Bff	PF	48-65		32.4	0.61	0.05
C	C1	O	A	0-22		49.5	15.40	1.02
	C2	A	A	22-48	Slightly sandy	31.8	5.96	0.46
	C3	B	B	48-55		18.3	1.39	0.10
	C4	Ojj	J	30-34	Located within the A horizon	39.8	8.40	0.65
	C5	Bff	PF	55-65		22.8	0.94	0.07
D	D1	O	O	0-30	Peat layer	72.8	27.09	1.28
	D2	B	B	30-37		23.2	1.98	0.12
	D3	Ajj	J	37-42		47.2	10.96	0.58
	D4	Ajj	J	45-50	Buried material located below the permafrost table	74.6	12.95	0.51
E	E1	O	O	0-35	Peat layer	81.2	31.64	1.50
	E2	B	B	35-40		31.6	4.74	0.31
	E3	Ojj	J	40-45	Hardly decomposed, coarse organic material	72.9	22.00	1.38
	E4	Bff	PF	46-49, and 54-65		24.7	0.80	0.07
	E5	Ojj	J	49-54	Well decomposed. fine black organic material, located below the permafrost table	76.2	22.02	1.40
F	F1	O	O	0-35	Peat layer	77.7	28.15	1.50
	F2	B	B	35-40		32.9	6.86	0.45
	F3	Bff	PF	42-60		41.5	4.09	0.26
	F4	Ojj	J	42-60	Buried material located below the permafrost table	79.5	21.06	1.41
G	G1	O	O	0-20	Only in depressions	44.2	17.40	0.83
	G2	B	B	0-40, or 20-40	Depth depending on the presence of the O horizon	22.3	2.89	0.19
	G3	Bff	PF	46-50		90.5	2.07	0.15
	G4	Ajj	J	10-15 or 35-40	Hardly decomposed, coarse organic material	33.6	9.14	0.50
	G5	Ajj	J	30-46	Well decomposed, fine black organic material, no visible plant remains	41.4	10.3	0.65
H	H1	O	O	0-15		41.7	17.63	0.82
	H2	B	B	15-30, and 40-42		18.0	1.69	0.12
	H3	Ajj	J	30-40	Well decomposed, fine black organic material	30.1	6.15	0.38
	H4	Bff	PF	42-50		91.9	2.71	0.18

* Soil horizons were defined on-site and their classification into soil groups was verified by determination of total organic carbon (TOC). Distinction of O and A horizons: O horizon > 17% TOC, A horizon < 17% TOC. Buried O (Ojj) and A (Ajj) horizons were grouped into (J). Classification of soil horizons follows the USDA Soil Taxonomy.

Table S3. Taxonomic distribution of bacterial and archaeal SSU rRNA amplicon reads (relative abundance, %).

Site 1

Phylum	Class	ZKA1	ZKA2	ZKA3	ZKA4	ZKB1	ZKB2	ZKB4
	Sample ID	**ZKA1**	**ZKA2**	**ZKA3**	**ZKA4**	**ZKB1**	**ZKB2**	**ZKB4**
	Soil horizon	A	A	B	J	A	C	J
**Phylum**	**Class**							
Crenarchaeota	MBGA	0.00	0.00	0.00	0.00	0.00	0.00	0.00
	MBGB	0.00	0.00	0.00	0.00	0.00	0.00	0.00
	MCG	0.00	0.00	0.01	0.00	0.00	0.01	0.00
	Thaumarchaeota	0.00	0.06	0.21	0.01	0.00	0.28	0.00
Euryarchaeota	DSEG	0.00	0.00	0.00	0.00	0.00	0.00	0.00
	Halobacteria	0.00	0.00	0.00	0.00	0.00	0.00	0.00
	Methanobacteria	0.00	0.00	0.00	0.00	0.00	0.00	0.00
	Methanomicrobia	0.00	0.00	0.00	0.00	0.00	0.00	0.00
	Thermoplasmata	0.00	0.00	0.00	0.00	0.00	0.00	0.00
[Parvarcheota]	*[Parvarchaea]*	0.00	0.00	0.00	0.00	0.00	0.05	0.00
Acidobacteria	Unclassified	0.07	0.22	0.04	0.12	0.05	0.05	0.07
	AT-s54	0.00	0.01	0.00	0.01	0.01	0.00	0.00
	Acidobacteria-5	0.05	0.20	0.00	0.15	0.07	0.02	0.05
	Acidobacteria-6	**3.73**	**6.79**	**4.74**	**6.27**	**3.57**	**5.73**	**3.73**
	Acidobacteria	**3.97**	**2.37**	**3.51**	**2.95**	**5.01**	**2.09**	**3.97**
	BPC102	0.00	0.00	0.00	0.00	0.01	0.27	0.00
	DA052	**1.83**	**1.31**	0.07	**1.61**	**1.68**	0.36	**1.83**
	EC1113	0.01	0.02	0.00	0.04	0.01	0.11	0.01
	Holophagae	0.04	0.02	0.12	0.04	0.93	0.06	0.04
	OS-K	0.00	0.00	0.00	0.00	0.00	0.00	0.00
	PAUC37f	0.00	0.01	0.00	0.01	0.01	0.01	0.00
	RB25	0.02	0.02	0.00	0.02	0.03	0.02	0.02
	S035	0.00	0.00	0.00	0.00	0.00	0.00	0.00
	Solibacteres	**3.36**	**2.72**	0.88	**2.52**	**3.56**	**1.69**	**3.36**
	Sva0725	0.12	0.09	0.00	0.06	0.23	0.15	0.12
	TM1	0.03	0.06	0.00	0.03	0.03	0.24	0.03
	[Chloracidobacteria]	**4.27**	**5.76**	**2.02**	**4.45**	**3.10**	**5.26**	**4.27**
	iii1-8	**2.56**	**3.17**	**2.57**	**3.02**	**3.53**	**3.49**	**2.56**
Actinobacteria	*Unclassified*	0.00	0.00	0.00	0.00	0.00	0.00	0.00
	Acidimicrobia	**1.20**	**1.71**	**1.78**	**1.71**	**1.61**	**1.22**	0.01
	Actinobacteria	**3.20**	**1.81**	**5.63**	**1.95**	**3.39**	**3.92**	**3.87**
	Coriobacteria	0.00	0.00	0.13	0.00	0.00	0.00	**3.94**
	MB-A2-108	0.03	0.19	0.19	0.08	0.04	0.39	0.00
	OPB41	0.00	0.01	0.09	0.04	0.04	0.13	0.00
	Rubrobacteria	0.00	0.00	0.00	0.00	0.00	0.00	0.00
	Thermoleophilia	**1.32**	**3.74**	**5.41**	**2.37**	**2.65**	**6.13**	0.01
Armatimonadetes	0319-6E2	0.03	0.04	0.00	0.02	0.01	0.00	0.00
	Armatimonadia	0.09	0.03	0.00	0.03	0.14	0.03	0.00
	Chthonomonadetes	0.24	0.14	0.14	0.13	0.18	0.08	0.00
	OPB50	0.00	0.00	0.00	0.00	0.00	0.00	0.00
	SHA-37	0.00	0.00	0.00	0.00	0.00	0.01	0.00
	SJA-176	0.00	0.00	0.14	0.00	0.01	0.39	0.00
	[Fimbriimonadia]	0.24	0.09	0.24	0.12	0.28	0.06	0.00
Bacteroidetes	Unclassified	0.00	0.00	0.00	0.00	0.00	0.00	0.00
	At12OctB3	0.00	0.00	0.00	0.00	0.00	0.00	0.00
	BME43	0.00	0.00	0.00	0.00	0.00	0.00	0.00
	Bacteroidia	0.01	0.06	**2.06**	0.16	0.04	**1.04**	**8.02**
	Cytophagia	0.36	0.05	0.10	0.07	0.09	0.01	0.00
	Flavobacteriia	0.02	0.03	0.14	0.02	0.01	0.00	0.01
	Sphingobacteria	0.86	1.00	**1.99**	0.90	0.65	0.84	0.03
	VC2_1_Bac22	0.01	0.00	0.00	0.00	0.01	0.00	0.00
	[Rhodothermi]	0.00	0.00	0.00	0.00	0.00	0.00	0.00
	[Saprospirae]	**4.75**	**4.81**	**2.91**	**4.25**	**3.47**	**3.67**	0.18
Caldiserica	*Caldisericia*	0.00	0.00	0.00	0.00	0.00	0.00	0.00
	OP5	0.00	0.00	0.00	0.00	0.00	0.00	0.00
	WCHB1-03	0.00	0.00	0.10	0.00	0.00	0.00	0.00
Chlamydiae	*Chlamydiae*	0.02	0.01	0.00	0.02	0.01	0.01	0.00
Chlorobi	Unclassified	0.04	0.06	0.03	0.05	0.03	0.02	0.00
	BSV26	0.00	0.01	0.00	0.01	0.01	0.06	0.00
	Ignavibacteria	0.00	0.00	0.00	0.00	0.01	0.00	0.00
	OPB56	0.01	0.01	0.09	0.02	0.00	0.03	0.00
	SJA-28	0.09	0.08	0.04	0.08	0.11	0.04	0.00
Chloroflexi	Anaerolineae	0.69	0.68	0.85	0.64	0.74	**1.68**	0.01
	C0119	0.05	0.02	0.15	0.03	0.05	0.11	0.00
	Chloroflexi	0.32	0.21	0.08	0.20	0.18	0.34	0.00
	Dehalococcoidetes	0.00	0.00	0.00	0.00	0.00	0.02	0.00
	Ellin6529	**1.97**	**2.31**	**4.35**	**2.34**	**1.36**	**3.08**	0.01
	Gitt-GS-136	0.05	0.15	0.72	0.21	0.03	0.49	0.00
	Ktedonobacteria	0.00	0.01	0.00	0.00	0.00	0.05	0.00
	P2-11E	0.00	0.03	0.00	0.02	0.00	0.23	0.00
	S085	0.13	0.28	0.52	0.25	0.22	0.78	0.00
	SHA-26	0.00	0.00	0.00	0.01	0.01	0.08	0.00
	TK10	**1.07**	0.67	0.16	0.82	**1.16**	0.30	0.00
	TK17	0.01	0.03	0.04	0.03	0.02	0.05	0.00
	Thermomicrobia	0.03	0.02	0.00	0.02	0.02	0.04	0.00
Cyanobacteria	4C0d-2	0.09	0.08	0.00	0.07	0.14	0.10	0.00
	Chloroplast	0.07	0.04	0.57	0.03	0.10	0.04	**9.35**
	Gloeobacterophycideae	0.00	0.00	0.00	0.00	0.00	0.00	0.00
	ML635J-21	0.02	0.05	0.00	0.04	0.03	0.03	0.00
	Nostocophycideae	0.00	0.00	0.00	0.00	0.00	0.00	0.01
	Oscillatoriophycideae	0.00	0.00	0.00	0.00	0.00	0.00	0.00
	Synechococcophycideae	0.00	0.00	0.00	0.00	0.00	0.00	0.00
Elusimicrobia	Unclassified	0.00	0.00	0.00	0.00	0.00	0.00	0.00
	4-29	0.00	0.00	0.00	0.00	0.00	0.00	0.00
	Elusimicrobia	0.53	0.56	0.34	0.52	0.74	0.31	0.01
	Endomicrobia	0.00	0.01	0.00	0.00	0.01	0.00	0.00
	OP2	0.00	0.00	0.00	0.00	0.00	0.00	0.00
Fibrobacteres	Fibrobacteria	0.03	0.05	0.37	0.12	0.01	0.03	0.00
	TG3	0.00	0.00	0.00	0.00	0.00	0.00	0.00
Firmicutes	*Bacilli*	0.03	0.03	**1.33**	0.03	0.02	0.17	**9.54**
	Clostridia	0.07	0.06	**1.05**	0.11	0.07	0.91	0.82
	Erysipelotrichi	0.00	0.00	0.24	0.00	0.00	0.16	0.00
Fusobacteria	*Fusobacteria*	0.00	0.00	0.00	0.00	0.00	0.00	0.00
Gemmatimonadetes	Gemm-1	0.39	0.94	0.42	0.85	0.37	0.43	0.00
	Gemm-2	0.00	0.02	0.26	0.01	0.01	0.00	0.00
	Gemm-3	0.00	0.00	0.17	0.00	0.00	0.00	0.00
	Gemm-5	0.00	0.03	0.01	0.02	0.00	0.05	0.00
	Gemmatimonadetes	**2.13**	**5.36**	**5.10**	**3.89**	**1.89**	**9.48**	0.01
Lentisphaerae	*Lentisphaerae*	0.00	0.00	0.00	0.00	0.00	0.00	0.00
Nitrospira	*Nitrospira*	0.50	**1.30**	**1.04**	**1.04**	0.93	**1.50**	**4.62**
Planctomycetes	Unclassified	0.00	0.01	0.00	0.01	0.01	0.00	0.00
	028H05-P-BN-P5	0.01	0.00	0.00	0.00	0.01	0.01	0.00

	BD7-11	0.04	0.01	0.00	0.03	0.06	0.00	0.00
	C6	0.00	0.01	0.00	0.00	0.00	0.00	0.00
	OM190	0.15	0.17	0.00	0.16	0.12	0.00	0.00
	Phycisphaerae	0.93	0.87	0.59	0.81	0.88	0.71	0.01
	Pla3	0.00	0.01	0.00	0.00	0.00	0.00	0.00
	Pla4	0.05	0.00	0.00	0.01	0.02	0.00	0.00
	Planctomycetia	**1.82**	**1.02**	**1.80**	**1.14**	**1.57**	0.86	**3.17**
	vadinHA49	0.14	0.17	0.11	0.12	0.04	0.14	0.00
Proteobacteria	*Unclassified*	0.00	0.00	0.00	0.00	0.00	0.00	0.00
	Alphaproteobacteria	**16.43**	**12.15**	**9.11**	**14.91**	**16.43**	**5.24**	0.11
	Betaproteobacteria	**7.64**	**7.60**	**15.11**	**7.98**	**7.79**	**14.55**	**9.77**
	Deltaproteobacteria	**10.34**	**7.08**	**4.81**	**7.95**	**13.01**	**7.43**	**7.06**
	Epsilonproteobacteria	0.00	0.00	0.00	0.00	0.00	0.00	0.00
	Gammaproteobacteria	**3.95**	**2.16**	**2.97**	**3.06**	**3.44**	**1.48**	**11.30**
	TA18	0.07	0.08	0.00	0.09	0.12	0.00	0.00
	Zetaproteobacteria	0.03	0.06	0.02	0.08	0.04	0.03	0.00
Spirochaetes	GN05	0.00	0.00	0.00	0.00	0.00	0.00	0.00
	MVP-15	0.00	0.00	0.00	0.00	0.00	0.00	0.00
	Spirochaetes	0.00	0.00	0.00	0.00	0.00	0.01	0.00
	[Brevinematae]	0.00	0.00	0.00	0.00	0.00	0.00	0.00
	[Leptospirae]	0.00	0.00	0.00	0.00	0.00	0.00	0.00
Tenericutes	Mollicutes	0.00	0.00	**1.08**	0.00	0.00	0.12	**2.49**
Verrucomicrobia	Unclassified	0.00	0.00	0.00	0.00	0.00	0.00	0.00
	Opitutae	0.73	0.38	**1.93**	0.50	**1.14**	0.24	0.01
	Verruco-5	0.01	0.01	0.00	0.01	0.01	0.03	0.00
	Verrucomicrobiae	0.03	0.08	0.21	0.06	0.02	0.07	0.01
	[Methylacidiphilae]	0.32	**1.25**	0.13	**1.05**	0.38	0.06	0.01
	[Pedosphaerae]	**2.52**	**2.72**	**3.75**	**2.36**	**3.12**	**2.66**	0.01
	[Spartobacteria]	**13.41**	**13.68**	**3.97**	**14.18**	**8.16**	**5.65**	**9.14**
[Thermi]	*Deinococci*	0.00	0.00	0.00	0.00	0.00	0.00	0.00
Unclassified bacteria		0.00	0.00	0.00	0.00	0.00	0.00	0.00
AC1	Unclassified	0.00	0.00	0.00	0.00	0.00	0.00	0.00
	SHA-114	0.00	0.00	0.00	0.00	0.00	0.00	0.00
AD3	Unclassified	0.00	0.10	0.10	0.09	0.03	0.25	0.00
	ABS-6	0.07	0.10	0.31	0.12	0.09	**1.30**	0.00
	JG37-AG-4	0.01	0.01	0.00	0.02	0.05	0.01	0.00
BHI80-139	Unclassified	0.01	0.01	0.00	0.01	0.01	0.02	0.00
BRC1	PRR-11	0.02	0.01	0.05	0.01	0.01	0.01	0.00
FBP	Unclassified	0.03	0.05	0.18	0.05	0.02	0.10	0.00
FCPU426	Unclassified	0.00	0.03	0.18	0.01	0.01	0.00	0.00
GN02	3BR-5F	0.00	0.00	0.00	0.00	0.00	0.00	0.00
	BD1-5	0.00	0.00	0.00	0.00	0.00	0.00	0.00
	GKS2-174	0.00	0.00	0.00	0.00	0.00	0.00	0.00
GN04	Unclassified	0.00	0.00	0.00	0.00	0.00	0.00	0.00
	MSB-5A5	0.00	0.00	0.00	0.00	0.00	0.00	0.00
GOUTA4	Unclassified	0.00	0.00	0.00	0.00	0.00	0.00	0.00
LD1	Unclassified	0.00	0.00	0.00	0.00	0.00	0.00	0.00
MVP-21	Unclassified	0.00	0.00	0.00	0.00	0.00	0.00	0.00
NC10	Unclassified	0.00	0.00	0.00	0.00	0.00	0.00	0.00
	12-24	0.00	0.00	0.00	0.00	0.00	0.00	0.00
NKB19	Unclassified	0.00	0.00	0.00	0.00	0.00	0.00	0.00
	TSBW08	0.00	0.00	0.00	0.00	0.00	0.00	0.00
OD1	Unclassified	0.01	0.00	0.00	0.00	0.01	0.03	0.00
	ABY1	0.00	0.00	0.00	0.00	0.00	0.00	0.00
	Mb-NB09	0.00	0.00	0.00	0.00	0.00	0.00	0.00
	SM2F11	0.07	0.10	0.18	0.10	0.09	0.23	0.00
	ZB2	0.01	0.02	0.01	0.02	0.03	0.04	0.00
OP11	Unclassified	0.00	0.00	0.00	0.00	0.00	0.00	0.00
	OP11-2	0.00	0.00	0.00	0.00	0.00	0.00	0.00
	OP11-3	0.00	0.00	0.00	0.00	0.00	0.01	0.00
	OP11-4	0.00	0.00	0.00	0.00	0.00	0.00	0.00
	WCHB1-64	0.00	0.00	0.00	0.00	0.00	0.00	0.00
OP3	BD4-9	0.00	0.00	0.00	0.00	0.00	0.00	0.00
	PBS-25	0.01	0.01	0.00	0.00	0.00	0.00	0.00
	koll11	0.02	0.05	0.00	0.05	0.01	0.01	0.00
OP8	OP8_1	0.00	0.00	0.00	0.00	0.00	0.00	0.01
	OP8_2	0.00	0.00	0.00	0.00	0.00	0.00	0.00
OP9	JS1	0.00	0.00	0.00	0.00	0.00	0.00	0.00
SC4	Unclassified	0.00	0.00	0.00	0.00	0.00	0.00	0.00
SR1	Unclassified	0.00	0.00	0.00	0.00	0.00	0.00	0.00
TM6	SBRH58	0.00	0.00	0.00	0.00	0.00	0.00	0.00
	SJA-4	0.01	0.01	0.00	0.02	0.02	0.00	0.00
TM7	Unclassified	0.00	0.00	0.00	0.00	0.00	0.00	0.00
	MJK10	0.00	0.01	0.00	0.00	0.00	0.01	0.00
	SC3	0.10	0.12	0.14	0.08	0.21	0.07	0.00
	TM7-1	0.01	0.01	0.02	0.01	0.01	0.00	0.00
	TM7-3	0.00	0.00	0.00	0.00	0.00	0.00	0.00
TPD-58	Unclassified	0.00	0.00	0.00	0.00	0.00	0.00	0.00
WPS-2	Unclassified	0.13	0.06	0.00	0.12	0.21	0.05	0.00
WS1	Unclassified	0.00	0.00	0.00	0.00	0.00	0.02	0.00
WS2	Kazan-3B-09	0.00	0.00	0.00	0.00	0.00	0.00	0.00
	SHA-109	0.01	0.01	0.00	0.01	0.01	0.00	0.00
WS3	PRR-12	0.06	0.10	0.07	0.08	0.06	0.07	0.00
WS4	Unclassified	0.00	0.00	0.00	0.00	0.00	0.01	0.00
WS5	Unclassified	0.00	0.00	0.00	0.00	0.00	0.00	0.00
ZB3	Unclassified	0.00	0.00	0.00	0.00	0.00	0.00	0.00

Table S3. Continued.

Site 2	Sample ID	ZKD1	ZKD2	ZKD3	ZKE2	ZKE3	ZKE4	ZKE5
	Soil horizon	O	B	J	B	J	PF	J
**Phylum**	**Class**							
Crenarchaeota	MBGA	0.00	0.00	0.00	0.00	0.00	0.00	0.00
	MBGB	0.00	0.00	0.00	0.00	0.00	0.00	0.00
	MCG	0.33	0.00	0.04	0.08	0.14	0.00	0.28
	Thaumarchaeota	0.00	0.00	0.00	0.02	0.00	0.00	0.00
Euryarchaeota	DSEG	0.00	0.00	0.00	0.00	0.00	0.00	0.00
	Halobacteria	0.00	0.00	0.00	0.00	0.00	0.00	0.00
	Methanobacteria	0.78	0.00	0.04	0.08	0.31	0.00	1.26
	Methanomicrobia	0.48	0.71	0.01	0.32	0.29	0.00	0.30
	Thermoplasmata	0.06	0.00	0.00	0.00	0.01	0.00	0.03
[Parvarcheota]	*[Parvarchaea]*	0.03	0.00	0.00	0.00	0.00	0.00	0.00
Acidobacteria	Unclassified	0.04	0.00	0.00	0.00	0.00	0.00	0.00
	AT-s54	0.00	0.00	0.00	0.00	0.00	0.00	0.00
	Acidobacteria-5	0.00	0.00	0.00	0.00	0.00	5.11	0.00
	Acidobacteria-6	1.72	0.63	1.54	4.58	2.77	3.43	2.25
	Acidobacteria	0.33	4.18	2.11	15.66	0.54	3.67	2.45
	BPC102	0.07	0.00	0.00	0.00	0.00	0.00	0.07
	DA052	0.09	1.98	0.42	0.56	0.12	0.00	0.26
	EC1113	0.00	0.21	0.06	0.18	0.07	0.00	0.04
	Holophagae	2.73	0.00	0.07	0.10	0.00	0.00	0.13
	OS-K	0.07	0.00	0.00	0.00	0.02	0.00	1.21
	PAUC37f	0.00	0.00	0.00	0.00	0.00	0.00	0.00
	RB25	0.00	0.00	0.00	0.00	0.00	0.00	0.00
	S035	0.00	0.00	0.00	0.00	0.00	0.00	0.00
	Solibacteres	3.13	0.94	9.82	3.18	5.75	5.83	2.80
	Sva0725	0.14	0.00	0.00	0.01	0.10	0.00	0.00
	TM1	0.03	0.09	0.01	0.03	0.00	0.00	0.17
	[Chloracidobacteria]	0.43	10.91	0.31	0.01	0.14	0.00	0.18
	iii1-8	2.37	2.14	4.44	4.59	2.81	0.00	11.33
Actinobacteria	*Unclassified*	0.02	0.00	0.00	0.02	0.18	0.00	0.15
	Acidimicrobia	0.84	0.00	0.27	0.12	0.48	0.00	1.29
	Actinobacteria	4.54	8.65	14.75	4.16	14.74	5.36	6.66
	Coriobacteria	0.00	0.62	0.01	0.01	0.20	0.48	0.00
	MB-A2-108	0.00	0.00	0.01	0.00	0.00	0.00	0.00
	OPB41	0.55	0.37	1.08	1.14	1.31	0.00	1.71
	Rubrobacteria	0.00	0.00	0.00	0.00	0.00	0.00	0.00
	Thermoleophilia	0.84	0.92	4.28	1.41	6.08	0.12	5.50
Armatimonadetes	0319-6E2	0.00	0.00	0.00	0.00	0.00	0.00	0.00
	Armatimonadia	0.00	0.00	0.00	0.00	0.00	0.00	0.00
	Chthonomonadetes	0.01	0.00	0.00	0.00	0.00	0.00	0.00
	OPB50	0.00	0.00	0.00	0.00	0.00	0.00	0.00
	SHA-37	0.07	0.01	0.03	0.06	0.00	0.00	0.00
	SJA-176	0.30	0.59	0.12	0.56	0.38	0.00	0.04
	[Fimbriimonadia]	0.06	0.00	0.00	0.00	0.00	0.00	0.00
Bacteroidetes	Unclassified	0.00	0.00	0.00	0.00	0.00	0.00	0.00
	At12OctB3	0.01	0.00	0.00	0.00	0.00	0.00	0.00
	BME43	0.00	0.00	0.00	0.00	0.00	0.00	0.00
	Bacteroidia	11.14	3.55	11.87	6.80	9.22	0.22	5.17
	Cytophagia	0.08	0.00	0.00	0.01	0.20	0.00	0.10
	Flavobacteriia	0.03	0.00	0.00	0.01	0.03	0.00	0.01
	Sphingobacteria	0.27	0.01	0.16	0.01	0.43	0.00	0.08
	VC2_1_Bac22	0.00	0.00	0.00	0.00	0.00	0.00	0.00
	[Rhodothermi]	0.00	0.00	0.00	0.00	0.00	0.00	0.00
	[Saprospirae]	0.69	3.41	16.43	2.15	8.98	0.01	1.71
Caldiserica	*Caldisericia*	0.00	0.00	0.00	0.00	0.00	0.00	0.00
	OP5	0.00	0.00	0.00	0.00	0.00	0.00	0.00
	WCHB1-03	0.01	0.00	0.39	0.41	1.79	0.00	1.33
Chlamydiae	*Chlamydiae*	0.01	0.00	0.00	0.00	0.00	0.00	0.00
Chlorobi	Unclassified	0.00	0.00	0.00	0.00	0.00	0.00	0.00
	BSV26	1.10	0.10	0.05	0.00	0.00	0.00	0.01
	Ignavibacteria	0.11	0.00	0.00	0.02	0.00	0.00	0.02
	OPB56	0.05	0.00	0.00	0.00	0.00	0.00	0.00
	SJA-28	0.62	0.00	0.05	0.03	0.03	0.00	0.09
Chloroflexi	Anaerolineae	5.74	0.19	0.73	2.95	4.37	0.00	1.13
	C0119	0.02	0.10	0.00	0.01	0.00	0.00	0.00
	Chloroflexi	0.12	0.00	0.00	0.00	0.00	0.00	0.00
	Dehalococcoidetes	0.05	0.01	0.03	0.01	0.01	0.00	0.00
	Ellin6529	2.44	1.14	2.73	3.05	3.17	0.01	1.36
	Gitt-GS-136	0.00	0.00	0.00	0.00	0.00	0.00	0.00
	Ktedonobacteria	0.00	0.00	0.00	0.00	0.00	0.00	0.00
	P2-11E	0.00	0.00	0.00	0.00	0.00	0.00	0.00
	S085	0.12	0.00	0.02	0.44	0.11	0.00	0.12
	SHA-26	0.02	0.00	0.00	0.01	0.00	0.00	0.00
	TK10	0.02	0.00	0.00	0.00	0.00	0.00	0.00
	TK17	0.02	0.00	0.00	0.04	0.00	0.00	0.00
	Thermomicrobia	0.01	0.02	0.00	0.03	0.01	0.02	0.00
Cyanobacteria	4C0d-2	0.03	0.00	0.01	0.00	0.04	0.00	0.00
	Chloroplast	0.09	1.53	0.00	0.03	0.08	4.84	0.06
	Gloeobacterophycideae	0.00	0.00	0.00	0.00	0.00	0.00	0.00
	ML635J-21	0.00	0.00	0.03	0.01	0.13	0.00	0.00
	Nostocophycideae	0.00	0.10	0.00	0.04	0.00	0.00	0.00
	Oscillatoriophycideae	0.00	0.77	0.00	0.02	0.00	0.00	0.00
	Synechococcophycideae	0.02	0.00	0.00	0.02	0.00	0.00	0.00
Elusimicrobia	Unclassified	0.00	0.00	0.00	0.00	0.00	0.00	0.00
	4-29	0.00	0.00	0.00	0.00	0.00	0.00	0.00
	Elusimicrobia	0.28	0.00	0.07	0.06	0.03	0.00	0.04
	Endomicrobia	0.04	0.00	0.00	0.00	0.00	0.00	0.00
	OP2	0.00	0.00	0.00	0.00	0.00	0.00	0.00
Fibrobacteres	Fibrobacteria	0.03	0.00	0.03	0.00	0.04	0.00	0.00
	TG3	0.08	0.59	0.02	0.02	0.00	0.00	0.00
Firmicutes	*Bacilli*	0.08	1.03	0.03	0.04	0.71	27.64	0.32
	Clostridia	2.49	4.03	2.00	3.34	5.25	5.43	4.16
	Erysipelotrichi	0.00	2.52	0.00	0.01	0.02	0.00	0.20
Fusobacteria	*Fusobacteria*	0.00	0.21	0.00	0.01	0.08	3.57	0.01
Gemmatimonadetes	Gemm-1	0.33	0.00	0.02	0.02	0.07	0.00	0.06
	Gemm-2	0.00	0.00	0.00	0.00	0.00	0.00	0.00
	Gemm-3	0.00	0.00	0.00	0.00	0.00	0.00	0.00
	Gemm-5	0.02	0.12	0.02	1.67	0.00	0.00	0.00
	Gemmatimonadetes	0.34	3.40	1.12	5.13	0.53	0.00	4.64
Lentisphaerae	*Lentisphaerae*	0.07	0.00	0.00	0.01	0.00	0.00	0.01
Nitrospira	*Nitrospira*	1.57	0.75	0.46	0.74	0.07	0.00	1.75
Planctomycetes	Unclassified	0.00	0.00	0.00	0.00	0.00	0.00	0.00
	028H05-P-BN-P5	0.02	0.00	0.00	0.00	0.00	0.00	0.00
	BD7-11	0.00	0.00	0.00	0.00	0.00	0.00	0.00
	C6	0.00	0.00	0.00	0.00	0.00	0.00	0.00
	OM190	0.02	0.00	0.00	0.00	0.00	0.00	0.05

	Phycisphaerae	0.38	0.84	0.07	0.10	0.36	0.00	0.58
	Pla3	0.03	0.00	0.00	0.00	0.00	0.00	0.00
	Pla4	0.00	0.00	0.00	0.00	0.00	0.00	0.00
	Planctomycetia	0.61	0.09	0.15	0.07	0.08	3.78	0.18
	vadinHA49	0.02	0.00	0.00	0.00	0.00	0.00	0.00
Proteobacteria	*Unclassified*	0.04	0.00	0.00	0.00	0.00	0.00	0.00
	Alphaproteobacteria	**5.41**	**3.41**	**4.43**	**1.68**	**6.31**	**5.38**	**16.71**
	Betaproteobacteria	**9.66**	**2.36**	**8.16**	**10.97**	**7.90**	0.35	**4.25**
	Deltaproteobacteria	**24.05**	**3.97**	**6.48**	**18.04**	**9.09**	**7.11**	**8.30**
	Epsilonproteobacteria	0.28	0.00	0.00	0.00	0.00	0.00	0.00
	Gammaproteobacteria	**1.74**	**29.78**	0.63	0.16	**1.68**	**7.22**	**2.05**
	TA18	0.03	0.00	0.00	0.00	0.00	0.00	0.00
	Zetaproteobacteria	0.00	0.00	0.00	0.00	0.00	0.00	0.00
Spirochaetes	GN05	0.01	0.00	0.00	0.00	0.00	0.00	0.00
	MVP-15	0.00	0.00	0.00	0.00	0.00	0.00	0.00
	Spirochaetes	0.32	0.00	0.01	0.04	0.00	0.00	0.00
	[Brevinematae]	0.02	0.00	0.00	0.00	0.03	0.00	0.00
	[Leptospirae]	0.00	0.00	0.00	0.01	0.00	0.00	0.12
Tenericutes	Mollicutes	0.00	0.00	0.00	0.04	0.00	0.00	0.00
Verrucomicrobia	Unclassified	0.00	0.00	0.00	0.00	0.00	0.00	0.00
	Opitutae	**1.17**	0.82	**1.01**	0.05	0.93	0.00	0.76
	Verruco-5	0.35	0.00	0.01	0.05	0.01	0.00	0.04
	Verrucomicrobiae	0.06	0.00	0.00	0.01	0.00	0.00	0.00
	[Methylacidiphilae]	0.33	0.00	0.02	0.01	0.00	0.00	**1.11**
	[Pedosphaerae]	**3.64**	**1.64**	**2.64**	**3.78**	**1.07**	0.00	**4.82**
	[Spartobacteria]	**1.22**	0.00	0.26	0.05	0.15	**2.99**	0.12
[Thermi]	*Deinococci*	0.00	0.00	0.00	0.00	0.00	0.00	0.00
Unclassified bacteria		0.00	0.00	0.00	0.00	0.00	0.00	0.00
AC1	Unclassified	0.00	0.00	0.00	0.00	0.00	0.00	0.00
	SHA-114	0.37	0.00	0.00	0.00	0.00	0.00	0.00
AD3	Unclassified	0.00	0.00	0.02	0.14	0.00	0.00	0.00
	ABS-6	0.00	0.28	0.00	0.11	0.00	0.00	0.00
	JG37-AG-4	0.00	0.00	0.00	0.04	0.00	0.00	0.00
BHI80-139	Unclassified	0.00	0.00	0.00	0.00	0.00	0.00	0.00
BRC1	PRR-11	0.07	0.00	0.01	0.01	0.00	0.00	0.00
FBP	Unclassified	0.01	0.00	0.01	0.00	0.05	**7.37**	0.01
FCPU426	Unclassified	0.04	0.00	0.01	0.00	0.00	0.00	0.00
GN02	3BR-5F	0.00	0.00	0.00	0.00	0.00	0.00	0.00
	BD1-5	0.00	0.00	0.00	0.00	0.00	0.00	0.00
	GKS2-174	0.00	0.00	0.00	0.00	0.00	0.00	0.00
GN04	Unclassified	0.00	0.00	0.00	0.00	0.00	0.00	0.00
	MSB-5A5	0.00	0.00	0.00	0.00	0.00	0.00	0.00
GOUTA4	Unclassified	0.00	0.00	0.00	0.00	0.00	0.00	0.00
LD1	Unclassified	0.00	0.00	0.00	0.00	0.00	0.00	0.00
MVP-21	Unclassified	0.00	0.00	0.00	0.00	0.00	0.00	0.00
NC10	Unclassified	0.00	0.00	0.00	0.00	0.00	0.00	0.00
	12-24	0.14	0.00	0.00	0.02	0.00	0.00	0.00
NKB19	Unclassified	0.00	0.00	0.00	0.00	0.00	0.00	0.00
	TSBW08	0.01	0.00	0.00	0.00	0.00	0.00	0.00
OD1	Unclassified	0.09	0.00	0.01	0.08	0.03	0.00	0.05
	ABY1	0.02	0.00	0.01	0.01	0.00	0.00	0.00
	Mb-NB09	0.00	0.00	0.00	0.00	0.00	0.00	0.00
	SM2F11	0.01	0.00	0.01	0.00	0.00	0.00	0.00
	ZB2	0.02	0.00	0.01	0.01	0.00	0.00	0.00
OP11	Unclassified	0.00	0.00	0.00	0.00	0.00	0.00	0.00
	OP11-2	0.00	0.00	0.01	0.01	0.03	0.00	0.00
	OP11-3	0.03	0.00	0.00	0.00	0.00	0.00	0.00
	OP11-4	0.00	0.00	0.00	0.00	0.00	0.00	0.00
	WCHB1-64	0.01	0.00	0.00	0.00	0.00	0.00	0.00
OP3	BD4-9	0.10	0.00	0.00	0.00	0.00	0.00	0.00
	PBS-25	0.30	0.00	0.00	0.00	0.00	0.00	0.00
	koll11	0.05	0.00	0.00	0.00	0.01	0.00	0.00
OP8	OP8_1	0.24	0.00	0.00	0.01	0.00	0.00	0.00
	OP8_2	0.12	0.00	0.00	0.32	0.00	0.00	0.00
OP9	JS1	0.00	0.00	0.00	0.01	0.00	0.00	0.00
SC4	Unclassified	0.02	0.00	0.00	0.00	0.00	0.00	0.00
SR1	Unclassified	0.01	0.00	0.00	0.00	0.00	0.00	0.00
TM6	SBRH58	0.00	0.00	0.00	0.00	0.00	0.00	0.00
	SJA-4	0.02	0.00	0.00	0.00	0.00	0.00	0.03
TM7	Unclassified	0.00	0.00	0.00	0.00	0.00	0.00	0.00
	MJK10	0.00	0.00	0.00	0.00	0.00	0.00	0.00
	SC3	0.15	0.00	0.21	0.08	0.19	0.00	0.12
	TM7-1	0.00	0.00	0.01	0.00	0.00	0.00	0.01
	TM7-3	0.01	0.00	0.03	0.01	0.07	0.00	0.02
TPD-58	Unclassified	0.02	0.00	0.00	0.00	0.00	0.00	0.00
WPS-2	Unclassified	0.01	0.28	0.00	0.00	0.01	0.00	0.11
WS1	Unclassified	0.09	0.00	0.00	0.00	0.00	0.00	0.00
WS2	Kazan-3B-09	0.00	0.00	0.00	0.00	0.00	0.00	0.00
	SHA-109	0.00	0.00	0.00	0.01	0.00	0.00	0.00
WS3	PRR-12	0.18	0.00	0.00	0.02	0.04	0.01	0.05
WS4	Unclassified	0.06	0.00	0.01	0.01	0.05	0.00	0.00
WS5	Unclassified	0.12	0.00	0.01	0.01	0.00	0.00	0.00
ZB3	Unclassified	0.00	0.00	0.00	0.00	0.00	0.00	0.00

Table S3. Continued.

**Site 3**	Sample ID<br>Soil horizon	**ZKG1**<br>O	**ZKG4**<br>J	**ZKH1**<br>O	**ZKH2**<br>B	**ZKH3**<br>J	**ZKH4**<br>PF
**Phylum**	**Class**						
Crenarchaeota	MBGA	0.00	0.00	0.00	0.00	0.00	0.00
	MBGB	0.00	0.00	0.00	0.00	0.00	0.00
	MCG	0.00	0.01	0.01	0.00	0.00	0.00
	Thaumarchaeota	0.00	0.00	0.00	0.00	0.00	0.03
Euryarchaeota	DSEG	0.00	0.00	0.00	0.00	0.00	0.00
	Halobacteria	0.00	0.00	0.00	0.00	0.00	0.00
	Methanobacteria	0.00	0.00	0.00	0.00	0.00	0.00
	Methanomicrobia	0.00	0.00	0.00	0.00	0.00	0.00
	Thermoplasmata	0.00	0.00	0.00	0.00	0.00	0.00
[Parvarcheota]	*[Parvarchaea]*	0.00	0.00	0.20	0.00	0.00	0.00
Acidobacteria	Unclassified	0.12	0.00	0.00	0.00	0.08	0.03
	AT-s54	0.01	0.00	0.00	0.00	0.01	0.00
	Acidobacteria-5	0.12	0.86	0.39	0.49	0.16	0.08
	Acidobacteria-6	**6.53**	**6.46**	**4.51**	**1.03**	**5.59**	**3.93**
	Acidobacteria	**1.83**	**1.88**	**1.32**	**1.06**	**2.13**	**4.83**
	BPC102	0.00	0.00	0.00	0.00	0.00	0.01
	DA052	0.95	0.00	**1.22**	0.00	0.88	**1.87**
	EC1113	0.00	0.00	0.00	0.00	0.02	0.03
	Holophagae	0.00	0.00	0.21	0.00	0.01	0.38
	OS-K	0.00	0.00	0.00	0.00	0.00	0.00
	PAUC37f	0.00	0.00	0.00	0.00	0.00	0.00
	RB25	0.01	0.00	0.00	0.00	0.01	0.02
	S035	0.00	0.00	0.00	0.00	0.00	0.00
	Solibacteres	**1.98**	**2.65**	**2.00**	0.08	**1.77**	**3.74**
	Sva0725	0.17	0.00	0.00	0.00	0.15	0.15
	TM1	0.01	0.00	0.00	0.00	0.01	0.06
	[Chloracidobacteria]	**2.19**	**2.38**	**4.03**	**1.12**	**1.99**	**3.20**
	iii1-8	0.72	0.00	0.75	0.00	0.67	**3.95**
Actinobacteria	*Unclassified*	0.00	0.00	0.00	0.00	0.00	0.00
	Acidimicrobia	**1.85**	**2.02**	**1.46**	0.00	**2.38**	**1.29**
	Actinobacteria	**6.85**	**10.35**	**9.43**	**10.09**	**9.42**	**2.25**
	Coriobacteria	0.00	0.00	0.04	**1.67**	0.00	0.00
	MB-A2-108	0.03	0.22	0.65	0.00	0.02	0.12
	OPB41	0.00	0.00	0.00	0.00	0.00	0.03
	Rubrobacteria	0.00	0.00	0.00	0.00	0.01	0.00
	Thermoleophilia	**2.71**	**3.27**	**3.63**	**1.37**	**2.67**	**2.25**
Armatimonadetes	0319-6E2	0.03	0.00	0.00	0.00	0.03	0.01
	Armatimonadia	0.10	0.00	0.00	0.00	0.06	0.08
	Chthonomonadetes	0.18	0.55	0.06	0.00	0.11	0.16
	OPB50	0.00	0.00	0.00	0.00	0.00	0.00
	SHA-37	0.00	0.00	0.00	0.00	0.00	0.00
	SJA-176	0.00	0.00	0.00	0.00	0.00	0.01
	[Fimbriimonadia]	0.21	0.00	0.00	0.02	0.26	0.17
Bacteroidetes	Unclassified	0.00	0.00	0.00	0.00	0.00	0.00
	At12OctB3	0.00	0.00	0.00	0.00	0.00	0.00
	BME43	0.00	0.00	0.00	0.00	0.00	0.00
	Bacteroidia	0.02	**2.41**	0.63	**3.10**	0.03	0.06
	Cytophagia	0.51	0.00	0.97	0.70	0.41	0.21
	Flavobacteriia	0.07	0.00	0.29	1.70	0.03	0.02
	Sphingobacteria	**1.48**	0.58	**2.03**	**2.19**	**1.60**	0.88
	VC2_1_Bac22	0.00	0.00	0.00	0.00	0.00	0.00
	[Rhodothermi]	0.00	0.00	0.00	0.00	0.00	0.00
	[Saprospirae]	**6.88**	0.52	**2.54**	0.75	**6.27**	**5.02**
Caldiserica	*Caldisericia*	0.00	0.00	0.00	0.00	0.00	0.00
	OP5	0.00	0.00	0.00	0.00	0.00	0.00
	WCHB1-03	0.00	0.00	0.06	0.00	0.00	0.00
Chlamydiae	*Chlamydiae*	0.01	0.00	0.01	0.00	0.02	0.02
Chlorobi	Unclassified	0.03	0.00	0.01	0.00	0.02	0.06
	BSV26	0.00	0.00	0.00	0.00	0.00	0.02
	Ignavibacteria	0.00	0.00	0.00	0.00	0.00	0.01
	OPB56	0.00	0.00	0.00	0.00	0.01	0.01
	SJA-28	0.08	0.00	0.06	0.00	0.07	0.13
Chloroflexi	Anaerolineae	0.39	0.00	0.28	0.45	0.31	0.65
	C0119	0.05	0.18	0.00	0.00	0.03	0.04
	Chloroflexi	0.24	0.25	0.30	0.00	0.17	0.20
	Dehalococcoidetes	0.00	0.01	0.00	0.00	0.00	0.00
	Ellin6529	**1.35**	**2.73**	**2.45**	0.03	**1.65**	**1.47**
	Gitt-GS-136	0.07	0.00	0.15	0.00	0.06	0.10
	Ktedonobacteria	0.00	0.00	0.00	0.00	0.35	0.01
	P2-11E	0.00	0.07	0.00	0.00	0.00	0.02
	S085	0.05	0.00	0.21	**1.45**	0.06	0.24
	SHA-26	0.00	0.00	0.00	0.00	0.00	0.02
	TK10	0.39	0.00	0.70	0.24	0.41	0.77
	TK17	0.03	0.00	0.01	0.00	0.03	0.02
	Thermomicrobia	0.04	0.51	0.00	0.05	0.02	0.01
Cyanobacteria	4C0d-2	0.13	0.34	0.00	0.00	0.10	0.08
	Chloroplast	0.14	**4.23**	0.37	**3.78**	0.10	0.15
	Gloeobacterophycideae	0.00	0.00	0.00	0.00	0.00	0.00
	ML635J-21	0.04	0.00	0.27	0.00	0.05	0.03
	Nostocophycideae	0.00	0.00	0.00	0.00	0.00	0.01
	Oscillatoriophycideae	0.00	0.00	0.00	0.00	0.00	0.00
	Synechococcophycideae	0.00	0.00	0.00	0.00	0.00	0.03
Elusimicrobia	Unclassified	0.00	0.00	0.00	0.00	0.00	0.00
	4-29	0.00	0.00	0.00	0.00	0.00	0.00
	Elusimicrobia	0.50	0.00	0.08	0.52	0.45	0.74
	Endomicrobia	0.00	0.00	0.00	0.02	0.01	0.00
	OP2	0.00	0.00	0.00	0.00	0.00	0.00
Fibrobacteres	Fibrobacteria	0.03	0.00	0.23	0.00	0.03	0.04
	TG3	0.00	0.00	0.00	0.00	0.00	0.00
Firmicutes	*Bacilli*	0.02	**8.13**	**3.69**	**7.84**	0.02	0.02
	Clostridia	0.03	**3.48**	0.60	**5.75**	0.03	0.11
	Erysipelotrichi	0.00	0.00	0.63	**1.02**	0.00	0.00
Fusobacteria	*Fusobacteria*	0.00	0.00	0.02	**2.28**	0.00	0.00
Gemmatimonadetes	Gemm-1	0.73	0.33	**1.57**	0.00	0.64	0.37
	Gemm-2	0.01	0.00	0.00	0.00	0.01	0.02
	Gemm-3	0.00	0.00	0.00	0.00	0.00	0.00
	Gemm-5	0.01	0.00	0.00	0.00	0.00	0.02
	Gemmatimonadetes	**3.04**	**4.95**	**5.45**	**1.32**	**2.92**	**2.85**
Lentisphaerae	*Lentisphaerae*	0.00	0.00	0.00	0.00	0.00	0.00
Nitrospira	*Nitrospira*	0.08	0.34	0.65	0.00	0.03	1.04
Planctomycetes	Unclassified	0.03	0.00	0.06	0.00	0.01	0.01
	028H05-P-BN-P5	0.00	0.00	0.00	0.00	0.00	0.01
	BD7-11	0.05	0.00	0.00	0.00	0.03	0.06
	C6	0.01	0.00	0.00	0.00	0.00	0.00
	OM190	0.20	0.00	0.20	0.00	0.10	0.12

	Phycisphaerae	**1.23**	**1.69**	**1.18**	0.12	**1.14**	0.85
	Pla3	0.00	0.00	0.00	0.00	0.00	0.00
	Pla4	0.01	0.00	0.00	0.00	0.01	0.01
	Planctomycetia	**1.55**	**2.32**	0.23	**2.42**	**1.07**	**1.04**
	vadinHA49	0.09	0.02	0.20	0.00	0.08	0.09
Proteobacteria	*Unclassified*	0.00	0.00	0.00	0.00	0.00	0.00
	Alphaproteobacteria	**18.85**	**14.11**	**12.45**	**14.98**	**21.84**	**14.69**
	Betaproteobacteria	**7.79**	**3.92**	**8.78**	**7.22**	**7.88**	**7.94**
	Deltaproteobacteria	**9.08**	0.27	**3.14**	**1.98**	**8.42**	**11.06**
	Epsilonproteobacteria	0.00	0.00	0.00	0.00	0.00	0.00
	Gammaproteobacteria	**4.41**	**1.31**	**4.76**	**17.13**	**4.77**	**3.76**
	TA18	0.07	0.00	0.01	0.00	0.08	0.09
	Zetaproteobacteria	0.00	0.00	0.00	0.00	0.00	0.02
Spirochaetes	GN05	0.00	0.00	0.00	0.00	0.00	0.00
	MVP-15	0.00	0.00	0.00	0.00	0.00	0.00
	Spirochaetes	0.00	0.00	0.00	0.00	0.00	0.00
	[Brevinematae]	0.00	0.00	0.00	0.00	0.00	0.00
	[Leptospirae]	0.00	**1.75**	0.00	0.00	0.00	0.00
Tenericutes	Mollicutes	0.00	0.00	0.04	0.00	0.00	0.00
Verrucomicrobia	Unclassified	0.01	0.00	0.00	0.00	0.01	0.00
	Opitutae	**1.29**	0.12	0.45	**1.62**	0.57	**1.43**
	Verruco-5	0.00	0.00	0.00	0.00	0.00	0.01
	Verrucomicrobiae	0.07	0.00	0.18	0.00	0.03	0.05
	[Methylacidiphilae]	0.35	0.37	0.21	0.00	0.25	0.36
	[Pedosphaerae]	**2.07**	0.00	**2.13**	**2.76**	**1.78**	**3.25**
	[Spartobacteria]	**9.30**	**10.23**	**11.11**	**1.56**	**7.04**	**9.85**
[Thermi]	*Deinococci*	0.00	0.00	0.06	0.00	0.00	0.00
Unclassified bacteria		0.01	0.00	0.00	0.00	0.00	0.00
AC1	Unclassified	0.00	0.00	0.00	0.00	0.00	0.00
	SHA-114	0.00	0.00	0.00	0.00	0.00	0.00
AD3	Unclassified	0.00	0.00	0.08	0.00	0.01	0.07
	ABS-6	0.02	0.00	0.28	0.00	0.01	0.34
	JG37-AG-4	0.00	0.00	0.00	0.00	0.01	0.04
BHI80-139	Unclassified	0.03	0.00	0.00	0.00	0.01	0.01
BRC1	PRR-11	0.01	0.00	0.00	0.00	0.01	0.03
FBP	Unclassified	0.06	0.00	0.00	0.00	0.07	0.04
FCPU426	Unclassified	0.00	0.00	0.00	0.00	0.00	0.00
GN02	3BR-5F	0.00	0.00	0.00	0.00	0.00	0.00
	BD1-5	0.00	0.00	0.00	0.00	0.00	0.00
	GKS2-174	0.00	0.00	0.00	0.00	0.00	0.00
GN04	Unclassified	0.00	0.00	0.00	0.00	0.00	0.00
	MSB-5A5	0.00	**1.28**	0.00	0.00	0.00	0.00
GOUTA4	Unclassified	0.00	0.00	0.00	0.00	0.00	0.00
LD1	Unclassified	0.00	0.00	0.00	0.00	0.00	0.00
MVP-21	Unclassified	0.00	0.00	0.00	0.00	0.00	0.00
NC10	Unclassified	0.00	0.00	0.00	0.00	0.00	0.00
	12-24	0.00	0.00	0.00	0.00	0.00	0.00
NKB19	Unclassified	0.00	0.00	0.00	0.00	0.00	0.00
	TSBW08	0.00	0.00	0.00	0.00	0.00	0.00
OD1	Unclassified	0.00	0.00	0.01	0.00	0.01	0.00
	ABY1	0.00	0.00	0.00	0.00	0.00	0.00
	Mb-NB09	0.00	0.00	0.00	0.00	0.00	0.00
	SM2F11	0.05	0.00	0.00	0.00	0.09	0.10
	ZB2	0.01	0.00	0.07	0.00	0.02	0.03
OP11	Unclassified	0.00	0.00	0.00	0.00	0.00	0.00
	OP11-2	0.00	0.00	0.00	0.00	0.00	0.00
	OP11-3	0.00	0.00	0.00	0.00	0.00	0.00
	OP11-4	0.00	0.00	0.00	0.00	0.00	0.00
	WCHB1-64	0.00	0.00	0.00	0.00	0.00	0.00
OP3	BD4-9	0.00	0.00	0.00	0.00	0.00	0.00
	PBS-25	0.00	0.00	0.00	0.00	0.00	0.00
	koll11	0.02	**1.90**	0.00	0.00	0.02	0.02
OP8	OP8_1	0.00	0.00	0.00	0.00	0.00	0.00
	OP8_2	0.00	0.00	0.00	0.00	0.00	0.00
OP9	JS1	0.00	0.00	0.00	0.00	0.00	0.00
SC4	Unclassified	0.00	0.00	0.00	0.00	0.00	0.00
SR1	Unclassified	0.00	0.00	0.00	0.00	0.00	0.00
TM6	SBRH58	0.00	0.00	0.00	0.00	0.00	0.00
	SJA-4	0.03	0.00	0.00	0.00	0.02	0.02
TM7	Unclassified	0.00	0.00	0.00	0.00	0.00	0.00
	MJK10	0.01	0.00	0.00	0.00	0.00	0.00
	SC3	0.08	0.44	0.04	0.00	0.10	0.14
	TM7-1	0.01	0.00	0.05	0.00	0.02	0.01
	TM7-3	0.00	0.00	0.00	0.02	0.00	0.00
TPD-58	Unclassified	0.00	0.00	0.00	0.00	0.00	0.00
WPS-2	Unclassified	0.06	0.00	0.00	0.00	0.04	0.15
WS1	Unclassified	0.00	0.00	0.00	0.00	0.00	0.00
WS2	Kazan-3B-09	0.00	0.00	0.00	0.00	0.00	0.00
	SHA-109	0.01	0.00	0.00	0.00	0.01	0.01
WS3	PRR-12	0.04	0.54	0.13	0.00	0.03	0.07
WS4	Unclassified	0.00	0.00	0.00	0.00	0.00	0.00
WS5	Unclassified	0.00	0.00	0.00	0.00	0.00	0.00
ZB3	Unclassified	0.00	0.00	0.00	0.00	0.00	0.00

Table S4. One-way ANOVA comparisons (P values) of extracellular enzyme activity potentials per gram dry soil in same soil horizons at the different sampling sites. P values < 0.05 are marked. CBH: 1,4-β-cellobiohydrolase, CHT: 1,4-β-poly-N-acetylglucosaminidase, NAG: β-N-acetylglucosaminidase, LAP: leucine aminopeptidase, POX: phenoloxidase, PER: peroxidase.

		**Hydrolytic enzymes**								**Oxidative enzymes**			
		CBH		CHT		NAG		LAP		POX		PER	
Horizon	Site	1	2	1	2	1	2	1	2	1	2	1	2
**O/A**	**2**	0.326		**0.000**		0.196		**0.037**		**0.002**		**0.002**	
	**3**	0.900	0.289	0.123	**0.013**	0.921	0.204	0.975	0.068	0.530	**0.002**	0.341	**0.002**
**B**	**2**	0.287		0.486		0.997		0.681		**0.004**		**0.002**	
	**3**	0.706	0.136	0.184	0.609	0.187	0.203	0.600	0.970	0.985	**0.006**	0.968	**0.003**
**J**	**2**	0.083		0.087		0.387		0.417		**0.002**		**0.003**	
	**3**	0.494	**0.017**	0.282	0.860	0.976	0.339	0.487	1.000	0.998	**0.003**	0.998	**0.005**
**PF**	**2**	1.000		0.841		0.736		0.973		0.212		0.125	
	**3**	0.107	0.136	**0.032**	0.067	0.499	0.911	**0.037**	0.059	0.389	0.860	0.455	0.538

Table S5. One-way ANOVA comparisons (P values) of extracellular enzyme activity potentials per gram organic carbon (OC) in same soil horizons at the different sampling sites. P values < 0.05 are marked. CBH: 1,4-β-cellobiohydrolase, CHT: 1,4-β-poly-N-acetylglucosaminidase, NAG: β-N-acetylglucosaminidase, LAP: leucine aminopeptidase, POX: phenoloxidase, PER: peroxidase.

		**Hydrolytic enzymes**								**Oxidative enzymes**			
		CBH		CHT		NAG		LAP		POX		PER	
Horizon	Site	1	2	1	2	1	2	1	2	1	2	1	2
**O/A**	**2**	0.370		**0.009**		0.929		0.359		0.084		0.204	
	**3**	0.280	0.926	0.144	0.390	0.342	0.548	0.385	0.096	0.057	**0.006**	**0.019**	**0.004**
**B**	**2**	0.148		0.93		**0.028**		**0.035**		**0.025**		0.128	
	**3**	0.445	**0.044**	0.057	0.081	**0.021**	0.769	0.186	0.535	0.946	**0.027**	0.982	0.139
**J**	**2**	**0.026**		0.491		0.999		0.073		0.052		0.067	
	**3**	0.679	**0.019**	0.195	0.578	0.556	0.514	0.631	0.536	0.633	**0.029**	0.669	**0.038**
**PF**	**2**	0.567		0.875		0.086		0.601		0.937		0.907	
	**3**	0.196	0.087	**0.014**	**0.014**	0.091	0.999	0.490	0.210	0.967	0.857	0.912	0.739
